# CRNKL1 Is a Highly Selective Regulator of Intron-Retaining HIV-1 and Cellular mRNAs

**DOI:** 10.1128/mBio.02525-20

**Published:** 2021-01-19

**Authors:** Han Xiao, Emanuel Wyler, Miha Milek, Bastian Grewe, Philipp Kirchner, Arif Ekici, Ana Beatriz Oliveira Villela Silva, Doris Jungnickl, Florian Full, Marco Thomas, Markus Landthaler, Armin Ensser, Klaus Überla

**Affiliations:** a Institute of Clinical and Molecular Virology, University Hospital Erlangen, Friedrich-Alexander Universität Erlangen-Nürnberg, Erlangen, Germany; b Berlin Institute for Medical Systems Biology, Max-Delbrück-Center for Molecular Medicine in the Helmholtz Association, Berlin, Germany; c Department of Molecular and Medical Virology, Ruhr-University, Bochum, Germany; d Institute of Human Genetics, University Hospital Erlangen, Friedrich-Alexander Universität Erlangen-Nürnberg, Erlangen, Germany; e IRI Life Sciences, Institute für Biologie, Humboldt Universität zu Berlin, Berlin, Germany; University of Pittsburgh; University of Pittsburgh School of Medicine

**Keywords:** CRNKL1, RNA splicing, association, human immunodeficiency virus, intron-retaining RNA, nuclear retention

## Abstract

The HIV-1 Rev protein is a nuclear export factor for unspliced and incompletely spliced HIV-1 RNAs. Without Rev, these intron-retaining RNAs are trapped in the nucleus. A genome-wide screen identified nine proteins of the spliceosome, which all enhanced expression from the HIV-1 unspliced RNA after CRISPR/Cas knockdown. Depletion of DHX38, WDR70, and four proteins of the Prp19-associated complex (ISY1, BUD31, XAB2, and CRNKL1) resulted in a more than 20-fold enhancement of unspliced HIV-1 RNA levels in the cytoplasm. Targeting of CRNKL1, DHX38, and BUD31 affected nuclear export efficiencies of the HIV-1 unspliced RNA to a much larger extent than splicing. Transcriptomic analyses further revealed that CRNKL1 also suppresses cytoplasmic levels of a subset of cellular mRNAs, including some with selectively retained introns. Thus, CRNKL1-dependent nuclear retention is a novel cellular mechanism for the regulation of cytoplasmic levels of intron-retaining HIV-1 mRNAs, which HIV-1 may have harnessed to direct its complex splicing pattern.

## Abstract (precis)

To regulate its complex splicing pattern, HIV-1 uses the adaptor protein Rev to shuttle unspliced or partially spliced mRNA from the nucleus to the cytoplasm. In the absence of Rev, these RNAs are retained in the nucleus, but it is unclear why.

## INTRODUCTION

Intron retention is increasingly recognized as a regulatory mechanism of gene expression and diversification of the cellular proteome (1). Transcriptomic analyses indicate that a large percentage of eukaryotic genes encode transcripts retaining introns (2, 3). Intron-retaining transcripts may be trapped in the nucleus, leading to their degradation. Alternatively, specialized nuclear export pathways may enable certain intron-retaining transcripts to reach the cytoplasm, where they can be translated or undergo nonsense-mediated decay. Retroviruses use intron retention to encode multiple proteins from one primary transcript. RNA secondary structures or cellular or viral adaptor proteins link intron-retaining retroviral transcripts in the nucleus to the TAP/NXF1 or the CRM-1 nuclear export factors (4), leading to the export of intron-retaining RNAs. While a number of studies have explored how nuclear retention can be overcome, the factors mediating nuclear retention of intron-containing mRNAs are mostly unknown but are likely linked to partial assembly of splicing complexes on intron-containing RNA (5).

The first evidence for a regulated export and expression of intron-retaining RNAs in mammalian cells was obtained in studies investigating the replication of human immunodeficiency virus type 1 (HIV-1). Alternative splicing and intron retention leads to three types of transcripts: fully spliced (FS) mRNAs, incompletely spliced (IS) mRNAs, and the unspliced (US) genomic RNA (6). The process of viral gene expression is temporally regulated, resulting in early expression of regulatory proteins and late expression of structural proteins (7–9). In the early phase, the US and IS transcripts are retained in the nucleus and are subjected to the nuclear splicing or degradation machinery (10). Only FS transcripts are transported to the cytoplasm via the default mRNA export pathway, TAP/NXF1, leading to expression of Tat, Rev, and Nef proteins (11, 12). The regulatory proteins Tat and Rev are nuclear cytoplasmic shuttle proteins and regulate viral gene expression. While Tat transactivates transcription by enhancing RNA elongation, Rev fine-tunes posttranscriptional processes and acts as a switch to initiate the late phase of the viral replication cycle (reviewed in references 13 and 14). It binds to the Rev-responsive element (RRE), which is present in the HIV-1 US and IS transcripts but not in the FS transcripts, and multimerizes on the RRE. The protein exporting factor CRM1 and associated factors are recruited by the Rev-RRE complex, leading to the nuclear-cytoplasmic export of these two classes of RNA (15).

Nuclear retention of intron-retaining HIV-1 RNAs has been hypothesized to be due to a number of mechanisms that are not mutually exclusive. One of them is the lack of *cis*-acting elements necessary for access to the TAP/NXF1 nuclear export pathway. Cell biological and virological studies have discovered that RNAs normally gain access to the exportin NXF1 by two means: either by recruitment of the transcription-export (TREX) complex via splicing (16) or by evolving particular regulatory sequences, e.g., the constitutive transport element (CTE) from type D retrovirus Mason-Pfizer monkey virus (MPMV), which has direct binding activity to NXF1 (17). A second explanation is the suboptimal splice sites of HIV that lead to the trapping of the intron-retaining RNAs in the spliceosome and subsequent degradation (18, 19). In addition, multiple inhibitory sequences (INS) were identified in the retained introns (20–24). The presumed inactivation of these inhibitory sequences by codon optimizing the AT-rich HIV-1 sequence abolished nuclear retention (25) independent of the presence or absence of HIV-1 splice donor (SD) and splice acceptor (SA) sites (26, 27). Why AT-rich intron-containing HIV-1 sequences, but not the codon-optimized variants, are retained remains elusive. It has been reported that knockdown of the paraspeckle long-noncoding RNA (lncRNA) NEAT1 could promote HIV Gag production through increased nucleocytoplasmic export of INS-containing RNAs, implicating the nuclear paraspeckles in the nuclear retention of HIV US and IS RNAs (28). However, this has been questioned by a study demonstrating that HIV-1 US RNA was not actually colocalized with paraspeckles (29), despite that several paraspeckle proteins (PSF, Martin3, and RBM14) have been reported to associate with HIV-1 INS elements and/or promote the Rev-dependent RNA export (30–34, 98).

In addition to these viral determinants and lncRNA contributing to nuclear mRNA retention, depletion of hnRNPA2/B1 has been shown to increase cytoplasmic levels of HIV-1 genomic RNA, but this increase did not enhance Gag expression levels and the precise mechanism remains unknown (35). With regard to nuclear retention of mRNA in general, a cellular complex named pre-mRNA retention and splicing complex (RES) has been identified in Saccharomyces cerevisiae (36). The RES is composed of Bud13, Snu17p, and Pml1P, and inactivation of any of the genes encoding these proteins in yeast reduces splicing of an intron with a weak 5′ splice donor. In addition, Pml1P inactivation enhanced expression of a pre-mRNA reporter construct without substantial reduction of the spliced mRNA, suggesting a role of Pml1P and the RES in nuclear retention. More recently, BUD13 was identified to bind to the poorly spliced mRNA of mammalian IRF7, a master regulator of the interferon response (37). Knockdown of BUD13 specifically enhanced retention of intron 4 of IRF7 in stimulated cells, thus reducing IRF7 protein levels and dampening the interferon response. However, intron-containing IRF7 mRNA could not be detected in the cytoplasm (37), indicating additional nuclear trapping mechanisms or rapid cytoplasmic degradation of intron-containing IRF7 mRNA. HIV-1 introns also differ from typical RES-dependent introns (37–39) by their low GC content, challenging the hypothesis that HIV-1 splicing may be regulated by the RES.

An alternative nuclear trapping mechanism could be a cellular quality control mechanism for mRNAs located at the nuclear pore complex (NPC). Experimental or stress-induced interference with this complex results in leakage of intron-retaining mRNAs into the cytoplasm (40–43). This finding suggests that the NPC-associated quality control complex also has a role in nuclear retention of intron-containing mRNAs, probably as a backup mechanism not influencing the initial splicing events. Here, we identified cellular proteins involved in nuclear trapping of intron-retaining HIV-1 mRNAs by performing a genome-wide screen for cellular factors, whose depletion resulted in enhanced expression from HIV-1 unspliced transcripts in the absence of its export factor Rev. Furthermore, we examined the effect of identified cellular factors on HIV US and FS RNA localization and expression. This revealed that CRNKL1 is a nuclear retention factor of the HIV-1 unspliced RNA and a selective regulator of cytoplasmic levels of a subset of intron-retaining cellular mRNAs.

## RESULTS

### Reporter cell lines for HIV-1 Rev-independent expression from the unspliced RNA.

**FIG 1:**
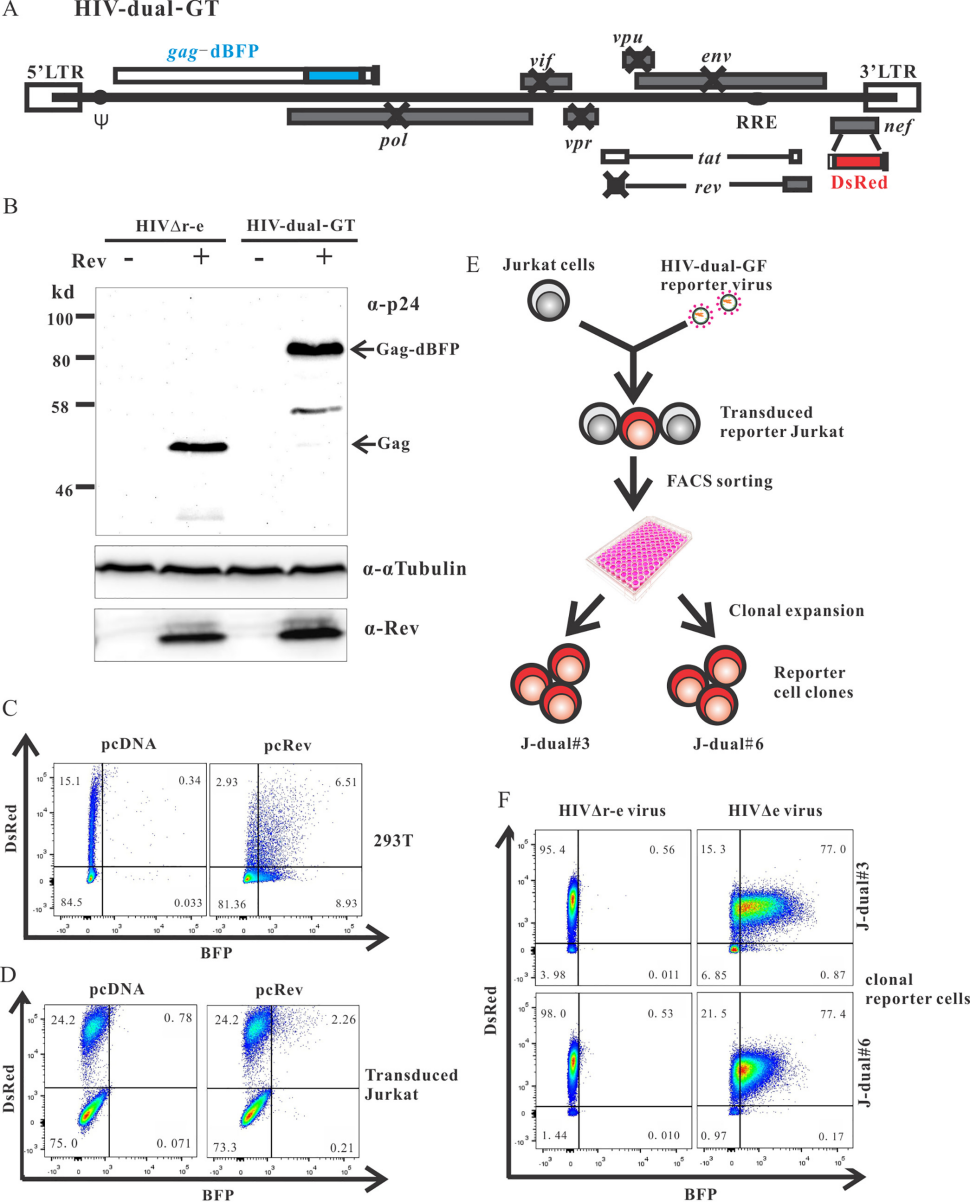
Establishment of HIV-dual-GT reporter cell clones. (A) Map of the HIV-dual-GT proviral reporter construct. (B) Western blot analysis of lysates of HEK293T cells transfected with HIV-dual-GT or HIV.r^−^e^−^ in the presence or absence of a Rev expression plasmid (pcRev) with antibodies to the p24 capsid protein, α-tubulin, or Rev. (C) 293T cells transfected with HIV-dual-GT and pcRev or pcDNA3.1 were analyzed by flow cytometry for Gag-BFP and DsRed expression. (D) Jurkat cells transduced with the HIV-dual-GT reporter vector (MOI, 0.3) and transiently transfected with pcRev or pcDNA control plasmid were analyzed by flow cytometry. (E) Scheme for the generation of reporter cell clones. (F) HIV-dual-GT-transduced Jurkat reporter cell clones J-dual#3 and J-dual#6 were superinfected with VSV-G-pseudotyped HIV-1 viruses differing only in the expression of Rev and analyzed by flow cytometry. All assays were performed at least twice, and results of one representative experiment are shown.

The overall strategy to identify cellular proteins involved in the nuclear trapping of intron-retaining HIV-1 mRNAs was to inactivate cellular genes by a genome-wide lentiviral CRISPR-Cas knockout library and to select knockout cells that show enhanced Gag expression from a proviral Rev-deficient HIV-1 reporter construct. To easily quantify HIV Gag expression in living cells, the Gag open reading frame (ORF) was fused to the blue fluorescent protein (BFP) gene. The *rev* gene was inactivated by two point mutations, and the DsRed reporter gene was expressed in place of *nef* from a fully spliced HIV-1 transcript in order to mark HIV-infected cells in the absence of Rev (Fig. 1A). Since we aimed to establish stable cell clones containing such proviral reporter constructs, expression of *pol*, *vif*, *vpr*, and *vpu* was also blocked by point mutations to avoid difficulties due to potential cytotoxic or cytostatic effects of these viral proteins (44–46). The *env* gene was inactivated by a 4-bp deletion, resulting in a frameshift. Although this reporter construct, designated HIV-dual-GT encodes only the Gag-BPF fusion protein, DsRed, and Tat (for sufficient transcriptional activation), all known *cis*-acting sequences potentially interacting with cellular components should be maintained.

To characterize the expression pattern of HIV-dual-GT, HEK293T cells were transfected with plasmids containing HIV-dual-GT or a *rev*-deficient *env* deletion mutant of HIV in the presence or absence of a Rev expression plasmid. Western blot analyses revealed Rev-dependent expression of the Gag-BFP fusion protein (Fig. 1B). Flow cytometric analysis of HEK293T cells transfected with HIV-dual-GT revealed a major DsRed-positive but BFP-negative cell population, which shifted to a double-positive population by cotransfection of a Rev expression plasmid (Fig. 1C). DsRed expression seemed to decrease in the presence of Rev, consistent with reduced splicing and consequently reduced generation of fully-spliced DsRed mRNA due to rapid export of unspliced transcripts (7).

To generate reporter cell lines harboring a properly integrated proviral DNA of HIV-dual-GT, it was encapsidated and pseudotyped by cotransfection with HIV-gag-pol and VSV-G expression plasmids. The Rev deficiency was also complemented by cotransfection of a Rev expression plasmid. Titers of the HIV-dual-GT vector were in the range of 5 × 10^5^ transforming units (TU)/ml. Flow cytometric analysis of Jurkat cells transduced with these VSV-G-pseudotyped HIV-dual-GT vector particles revealed a distinct population of cells that were DsRed positive but BFP negative (Fig. 1D). Transient transfection of this transduced bulk culture with a Rev expression plasmid shifted approximately 10% of the DsRed-positive population to a DsRed and BFP double-positive population. The magnitude of this effect was lower than in the cotransfection experiments in 293T cells (Fig. 1C), consistent with the lower transfection efficiency for Jurkat cells.

To obtain more homogenous reporter cells, Jurkat cell clones containing HIV-dual-GT were established by transducing Jurkat cells at a multiplicity of infection (MOI) of 0.1, followed by flow cytometric sorting of single DsRed-positive, BFP-negative cells into wells of a 96-well plate (Fig. 1E). Two of the expanded DsRed-positive cell clones, showing a rather homogenous population of DsRed-positive and BPF-negative cells and designated J-dual#3 and J-dual#6, were transduced at an MOI of 2 with VSV-G-peudotyped *env* deletion mutants of HIV containing or lacking a functional *rev* gene. In the presence of a functional *rev* gene, the BFP fluorescence was strongly induced in both reporter cell clones (Fig. 1F). Therefore, both clones were selected for the screening of cellular genes that suppress BFP expression from the HIV-dual-GT reporter virus.

### Genome-wide screening for cellular factors suppressing expression from intron-containing HIV-1 genomic RNA.

**FIG 2:**
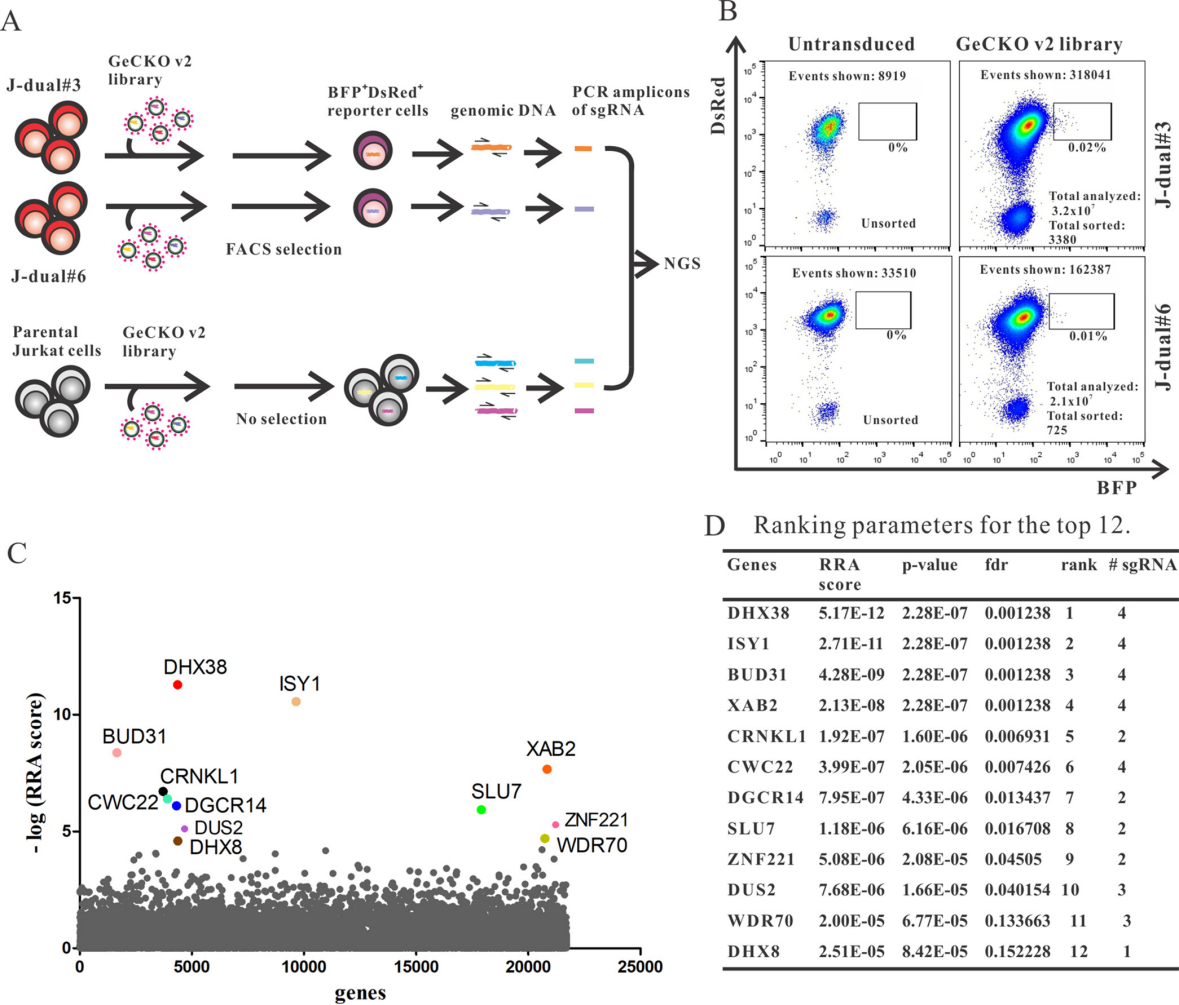
Genome-wide CRISPR/Cas screen. (A) Outline of screening strategy. (B) Flow cytometric analysis of J-dual#3 and J-dual#6 reporter cells without transduction and 4 days after transduction by the GeCKO v2 lentiviral vector library (MOI, around 2) immediately prior to the sorting process. The total number of events shown in the graph, total number of cells analyzed in the screen, and total numbers of cells sorted are indicated. (C) Enrichment analysis for guide RNAs in transduced and selected J-dual#3 and J-dual#6 reporter cells compared to transduced but unselected Jurkat cells by the MAGeCK program. The robust ranking aggregation (RRA) score reflects the enrichment of guide RNA targeting the same gene in cells selected from both reporter cell clones. The distribution of the negative log_10_ of the RRA score for all targeted genes in alphabetical order on the *x* axis is shown, with the top 12 candidate genes highlighted. (D) Additional MAGeCK ranking parameters of the top 12 candidate genes. fdr, false discovery rate; # sgRNA, number of enriched guide RNAs targeting the same gene.

Poor expression levels of HIV-1 structural genes in the absence of Rev that can be overcome by modifying the codon usage suggest that there is an active cellular suppressive mechanism acting on the level of the viral transcript. To identify cellular proteins necessary for this postulated suppressive mechanism, we used a GeCKOv2 genome-wide CRISPR-Cas knockout library kindly provided by the laboratory of Feng Zhang (47, 48). The plasmid library contains 122,411 different single guide RNA (sgRNA) genes targeting 19,050 human genes (6 sgRNAs per gene) and 1,864 sgRNA genes targeting microRNA (miRNA) genes (4 sgRNAs per miRNA gene) and includes 1,000 nontargeting (NT) sgRNA genes. Cas9, sgRNA, and puromycin resistance genes are expressed from a single lentiviral vector construct, lentiCRISPRv2. After amplification of the plasmid library and confirmation of its diversity by next-generation sequencing (NGS; data not shown), a stock of a VSV-G-pseudotyped lentiviral vector library was prepared by transient transfection of 293T cells and titrated on the Jurkat reporter cell clones, J-dual#3 and J-dual#6, and parental Jurkat cells. All three cell lines were then transduced with the lentiviral CRISPR library at an MOI of around 2. Four days after transduction, the 0.01 to 0.02% DsRed-positive cells showing the highest BFP expression levels were sorted for NGS of the sgRNA genes delivered to these cells by the lentiviral vector library. To be able to determine enrichment of sgRNA genes enhancing Gag-BFP expression levels, the representation of each sgRNA gene from nonselected Jurkat cells transduced with the lentiviral CRISPR library was also determined. Selective enrichment of sgRNA sequences in sorted J-dual#3 and J-dual#6 cells compared to nonsorted Jurkat cells was then determined by MAGeCK, a computational tool developed for the analysis of CRISPR screens (49, 50). The calculated robust ranking aggregation (RRA) score reflects enrichment of sgRNA sequences targeting the same gene in the selected cells. The distribution of the RRA score for all targeted genes is plotted in Fig. 2C, with the top 12 candidate genes highlighted. For 11 of these, at least two different sgRNA sequences targeting the same gene were enriched in the selected cells, arguing against confounding off-target effects of single sgRNA (Fig. 2D). The probability of false discovery of the top 12 candidate genes ranged from 0.0012 to 0.15.

### Analyzing interaction of validated hits shows a network of pre-mRNA splicing factors.

Based on the enrichment of the individual sgRNAs targeting each of the top 12 candidate genes in the Gag-BFP-positive cells selected by flow cytometry (see Fig. S1 in the supplemental material), two sgRNA sequences were chosen for each of the candidate genes (Table S1A) for further validation. Gene-specific sgRNA sequences and a nontargeting sequence, named NT1, were cloned individually into the lentiCRISPRv2 vector plasmid. J-dual#3 and J-dual#6 reporter cells were then transduced with lentiviral vector particles transferring these sgRNA sequences. Upregulation of BFP expression was observed by flow cytometry for 10 of the 12 candidate genes. For nine of them, transfer of both sgRNA sequences upregulated BFP expression in both reporter cell clones (Fig. S2A). For DUS2 and ZNF221, the screening results could not be confirmed. Since long half-lives of mRNAs and proteins may prolong the time from inactivation of the targeted gene to the decline of protein levels, we continued to monitor the reporter cells transduced with sgRNA genes targeting DUS2 and ZNF221 for up to 2 weeks postinfection. However, upregulation of Gag-BFP remained undetectable (data not shown). In addition, we confirmed sufficient transduction rates by the lentiviral DUS2 and ZNF221 targeting vectors, excluding the possibility that reduced lentiviral vector titers are responsible for their failure to upregulate BFP expression (Fig. S2B). Despite this, the entire screening approach was highly specific, showing a false discovery rate (FDR) of only 0.166.

10.1128/mBio.02525-20.1 FIG S1 Enrichment of individual sgRNAs targeting the top 12 candidate genes. NGS data from unselected Jurkat cells (control) and selected J-dual#3 and J-dual#6 cells were analyzed by MAGeCK. Results are shown as normalized counts for each of the six guide RNAs targeting the indicated candidate genes. Download FIG S1, TIF file, 1.2 MB. Copyright © 2021 Xiao et al. 2021 Xiao et al. This content is distributed under the terms of the Creative Commons Attribution 4.0 International license.

10.1128/mBio.02525-20.2 FIG S2 Validation of screening hits. (A) J-dual#3 and J-dual#6 reporter cells were coinfected with a lentiviral vector encoding Cas9 and a lentiviral vector expressing the indicated guide RNAs at an MOI of 1. Flow cytometric analyses were performed 7 days after infection. Each of the 12 candidate genes was targeted by two different sgRNAs. A nontargeting sgRNA (NT1) was used as a control. Representative results from at least two experiments are shown. (B) Percent transduction by individual lentiviral knockout vectors. All individual lentiCRISPR-KO constructs were packaged into VSV-G-pseudotyped lentiviral vector particles under the same conditions. The same volume of individual lentiCRISPR vector preparation was used to infect J-dual#3 cells. The percent transduction was calculated as described in Materials and Methods. Download FIG S2, TIF file, 2.0 MB. Copyright © 2021 Xiao et al. 2021 Xiao et al. This content is distributed under the terms of the Creative Commons Attribution 4.0 International license.

10.1128/mBio.02525-20.9 TABLE S1 Oligonucleotide sequences and primer combinations. (A) Sequences of sgRNAs used for validation of screening hits. (B) Sequences of primers used for construction of NGS libraries. (C) Primer combinations for construction of NGS libraries. (D) Sequences of primers used for validation of retained introns. (E) Sequences of the pooled siRNAs targeting CRNKL1. Download Table S1, TIF file, 1.5 MB. Copyright © 2021 Xiao et al. 2021 Xiao et al. This content is distributed under the terms of the Creative Commons Attribution 4.0 International license.

**FIG 3:**
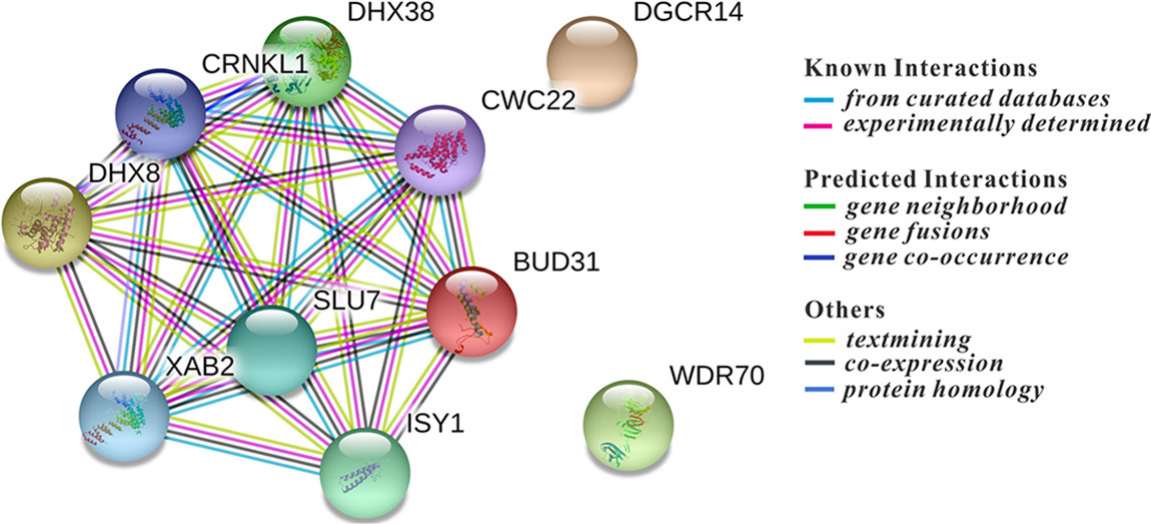
Protein interaction network of confirmed screening hits. The 10 validated hits were analyzed by the online database STRING for protein-protein interactions. The graph shows known and predicted protein-protein interactions for the most enriched pathway, the “mRNA splicing” pathway (pathway ID, GO 0000398; false discovery rate, 5.07e−14; minimum required interaction score, 0.400 [medium confidence]).

A first hint of potential mechanisms by which inactivation of the identified cellular genes could enhance Gag-BFP expression in the reporter cell lines was obtained by searching for functional protein association networks between the identified genes using the STRING online database (https://string-db.org/). Strikingly, 9 of the 10 confirmed hits are involved in pre-mRNA splicing (Fig. 3). Among them is ESS2/DGCR14, for which no interactions with the other identified factors have been reported but which is associated with U6 snRNA as well as U1 and U4 snRNAs (51). WDR70 is not linked to splicing; however, it contains a WD40 repeat domain which is shared by a number of splicing factors (52). The pre-mRNA splicing process includes two excision steps catalyzed by a large and dynamic RNP machine. The confirmed hits DHX38, DHX8, SLU7, and DGCR14 are typical second-step factors. In contrast, XAB2, ISY1, CRNKL1, CWC22, and BUD31 should be recruited at the first step, and intriguingly, all belong to the nuclear Prp19-associated complex (53–56). This complex is involved in diverse nuclear functions, such as DNA double-strand break (DSB) repair, transcriptional elongation, and splicing (57). The core factor of the complex, PRPF19 (human Prp19), however, was not identified in the screen. Consistently, targeting PRPF19 by two sgRNAs in J-dual#3 did not affect Gag-BFP expression (Fig. S3A). Since most of the confirmed hits are components of the spliceosome, we analyzed whether PRPF8, an ATPase/helicase at the core of the spliceosome (58), could also regulate the HIV Gag expression. Moreover, BUD13 from the RES complex and Tpr from the NPC, which were reported to have a nuclear retention function, were tested as well by using two different CRISPR/Cas sgRNAs targeting each of the genes. None of these targeting sgRNAs tested induced BFP expression (Fig. S3B to D).

10.1128/mBio.02525-20.3 FIG S3 Influence of selected proteins reported to be involved in pre-mRNA splicing and nuclear retention on Gag-BFP expression. Two sgRNAs for each gene were picked from the GeCKO v2 library to target PRPF8, PRPF19, TPR, and BUD13, respectively. The individual lentiCRISPR knockout vectors were constructed and used to transduce the J-dual#3 reporter cell line. Transduction efficiencies were confirmed (data not shown), and cells were analyzed by flow cytometry 7 days after transduction (A to D). Download FIG S3, TIF file, 0.6 MB. Copyright © 2021 Xiao et al. 2021 Xiao et al. This content is distributed under the terms of the Creative Commons Attribution 4.0 International license.

### CRNKL1 and other cellular factors impact HIV-1 unspliced and fully spliced mRNA levels.

**FIG 4:**
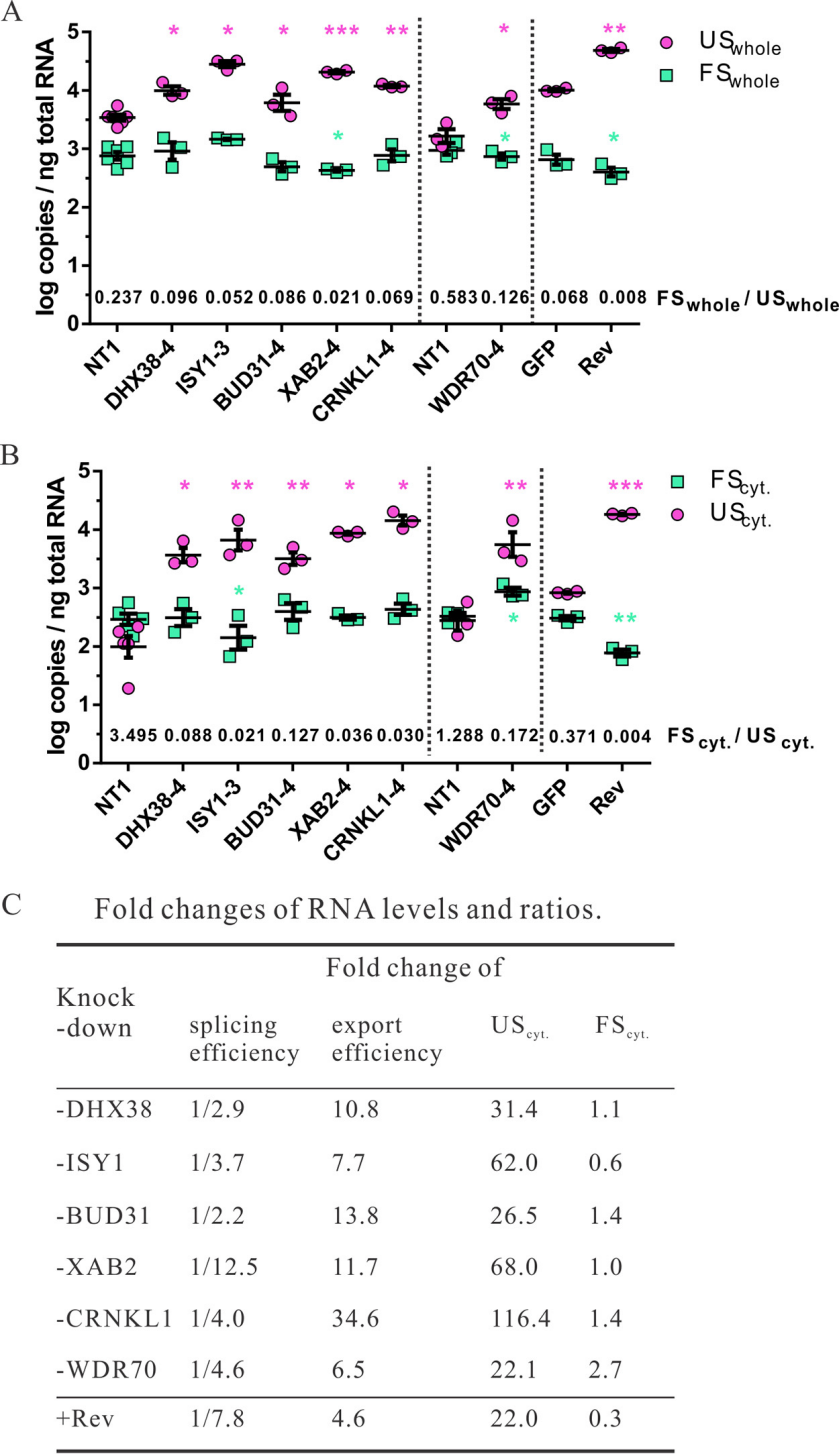
Role of confirmed screening hits in HIV-1 RNA splicing and export. (A) J-dual#3 cells were coinfected at an MOI of 3 with a lentiviral vector encoding Cas9 and a lentiviral vector expressing the guide RNAs indicated. In addition, J-dual#3 cells were transduced by a lentiviral vector encoding GFP or Rev at an MOI of 1. At 4 days after infection, BFP-positive cells were sorted by flow cytometry from cells transduced with lentiviral vectors encoding Rev or the guide RNAs targeting the confirmed hits. Matched controls expressing NT1 or GFP were used without sorting. Total RNA was purified from whole-cell extracts. HIV-1 US and FS RNA copy numbers in these samples were quantified by RT-qPCR and are shown as log copies per ng total RNA extracted from whole cells. Log copies from at least three experiments, as well as their mean and standard error of the mean (SEM), are shown. The mean of the FS_whole_/US_whole_ ratio for each experiment is shown above the *x* axis for each of the treatment groups. (B) J-dual#3 knockout cells and control cells were prepared as described for panel A, but total RNA was extracted from the cytoplasmic fraction of each cell sample. Log copies per ng total cytoplasmic RNA and mean FS_cyt._/US_cyt_ ratios. were calculated and presented as in panel A. (C) The fold changes in the different treatment groups for the splicing efficacy (FS_whole_/US_whole_), the nuclear export efficacy (US_cyt._/US_whole_), and cytoplasmic levels of unspliced (US_cyt_) and fully spliced (FS_cyt_) transcripts were calculated by dividing the values in the treatment groups by the ones obtained from the matched control cells (NT1, GFP) as described in Materials and Methods. A paired *t* test was performed on the log copy numbers/ng total RNA between each of the treatment groups and the matched control. *, *P* < 0.05; **, *P* < 0.01; ***, *P* < 0.001. Unless otherwise indicated, differences were not significant. Vertical dotted lines separate experiments that were not performed in parallel and therefore contain independent controls. WDR70.KD cells were sorted on day 7 after transduction, while other cells were sorted on day 4.

To follow up on the reduction of splicing as the potential mechanism of enhanced Gag-BFP expression, the top 5 hits and WDR70 were analyzed for their effect on HIV-1 mRNA splicing. Attempts to generate J-dual#3 cells with a stable knockout of the six selected target genes were not successful, presumably due to the fact that the selected genes are essential for cell viability (59). We therefore analyzed HIV RNA splicing and nuclear export after knockdown of these genes in short-term cultures. J-dual#3 cells were transduced with the lentiviral vectors encoding the different guide RNA genes, and Gag-BFP-positive cells were sorted to enrich for cells with the desired gene knockdown. Total RNA was then extracted from the sorted cells, and the copy numbers of unspliced HIV RNA per ng extracted total RNA were determined and compared to the copy numbers of unspliced RNA in J-dual#3 cells transduced with a negative control vector (NT1). Consistent with the stronger BFP expression, unspliced HIV RNA copy numbers were also enhanced 3- to 9-fold (Fig. 4A). Fully spliced HIV-1 RNAs were also quantified, revealing a marginal decrease in cells transduced with XAB2 and WDR70 targeting sgRNAs but not with the other gene targeting sgRNA (Fig. 4A). Differential effects of the gene targeting HIV-1 FS and HIV-1 US RNA also lead to decreased ratios of FS to US transcripts (Fig. 4A). For comparative reasons, we also transduced J-dual#3 cells in an independent experiment with a lentiviral vector expressing Rev, sorted Gag-BFP-positive cells, and quantified HIV-1 US and FS transcripts. Rev enhanced the US HIV-1 RNA levels approximately 5-fold, which coincided with a minor reduction in FS transcripts (Fig. 4A).

The splicing process is closely linked to nuclear mRNA trafficking (60, 61). Therefore, we also determined the effect of targeting the six selected candidate genes on the cytoplasmic levels of HIV-1 US and FS RNA (Fig. 4B). Proper separation of the cytoplasm from nuclear components was controlled by examining GAPDH (glyceraldehyde-3-phosphate dehydrogenase) pre-mRNA levels and a nuclear marker protein in the cytoplasmic fraction from Jurkat cells (62, 63). GAPDH pre-mRNA levels per ng total RNA extracted from the cytoplasm were approximately 50-fold lower than GAPDH pre-mRNA levels per ng total RNA extracted from whole cells. Consistently, the nuclear LaminB protein could not be detected in the cytoplasmic fractions (Fig. S4).

10.1128/mBio.02525-20.4 FIG S4 Validation of fractionation. (A) Representative Western blot analyses for LaminB (nuclear) and α-tubulin (cytoplasmic) in whole-cell extracts and nuclear and cytoplasmic fractions from 1 × 10^6^ Jurkat cells. (B) Differences in GAPDH pre-mRNA levels. Threshold cycle (*C_T_)* values obtained by real-time PCR from whole-cell or cytoplasmic extracts from three experiments are shown. Numbers indicate the fold difference between GAPDH pre-mRNA levels/weight of extracted RNA from whole-cell and cytoplasmic extracts, assuming 2-fold differences in the targeted RNA copy numbers per Δ*C_T_* and adjusting for minor differences in the amounts of RNA added to the PCR mixtures. Download FIG S4, TIF file, 0.2 MB. Copyright © 2021 Xiao et al. 2021 Xiao et al. This content is distributed under the terms of the Creative Commons Attribution 4.0 International license.

After transduction with the different targeting vectors, HIV-1 US RNA in the cytoplasm of Gag-BFP-positive cells was enhanced approximately 22- to 116-fold, while the cytoplasmic FS RNA levels were affected only 0.6- to 2.7-fold (Fig. 4B). The magnitude of the enhancement of cytoplasmic levels of HIV-1 US RNA exceeded the enhancement of HIV-1 US RNA in whole-cell extracts, indicating that knocking down the selected target genes not only reduced splicing but also enhanced nuclear export of the HIV-1 US RNA. Strong enhancement of cytoplasmic HIV-1 US RNA export was also observed in Rev-expressing cells. However, in contrast to the cells transduced with the targeting vectors, cytoplasmic FS RNA levels in Rev-transduced cells were significantly reduced, consistent with the previously reported suppression of the TAP/NXF1-mediated RNA export by Rev (64).

To dissect the relative contributions of inhibition of splicing and enhancement of nuclear export of HIV-1 US RNA, we compared the ratio of HIV-1 FS RNA to HIV-1 US RNA in whole-cell extracts as a measure of splicing efficiency and the ratio of HIV-1 US RNA in cytoplasmic extracts to the HIV-1 US RNA in whole-cell extracts as a measure of nuclear export efficiency. This revealed two different patterns of responses. The fold reduction in the splicing efficiencies in cells transduced with ISY1, XAB2, and WDR70 targeting vectors mirrored the fold enhancement of the respective nuclear export efficiencies. Expression of Rev induced a similar pattern of response. In contrast, transduction with DHX38, BUD31, or CRNKL1 targeting vectors predominantly enhanced the nuclear export efficiency with at least 3-fold-lower effects on splicing efficiency (Fig. 4C). In particular, knockdown of CRNKL1 enhanced the ratio of HIV-1 US RNA in cytoplasmic extracts to HIV-1 US RNA in total cell extracts more than 34-fold, while the ratio of HIV-1 FS RNA to HIV-1 US RNA in whole-cell extracts was decreased only 4-fold. Targeting of CRNKL1 also led to the strongest enhancement of cytoplasmic levels of HIV-1 US RNAs without reducing cytoplasmic HIV-1 FS RNA levels (Fig. 4C), indicating that CRNKL1 is a major nuclear retention factor of the genomic HIV-1 US RNA.

### CRNKL1 associates with HIV-1 US RNA in the nucleus.

**FIG 5:**
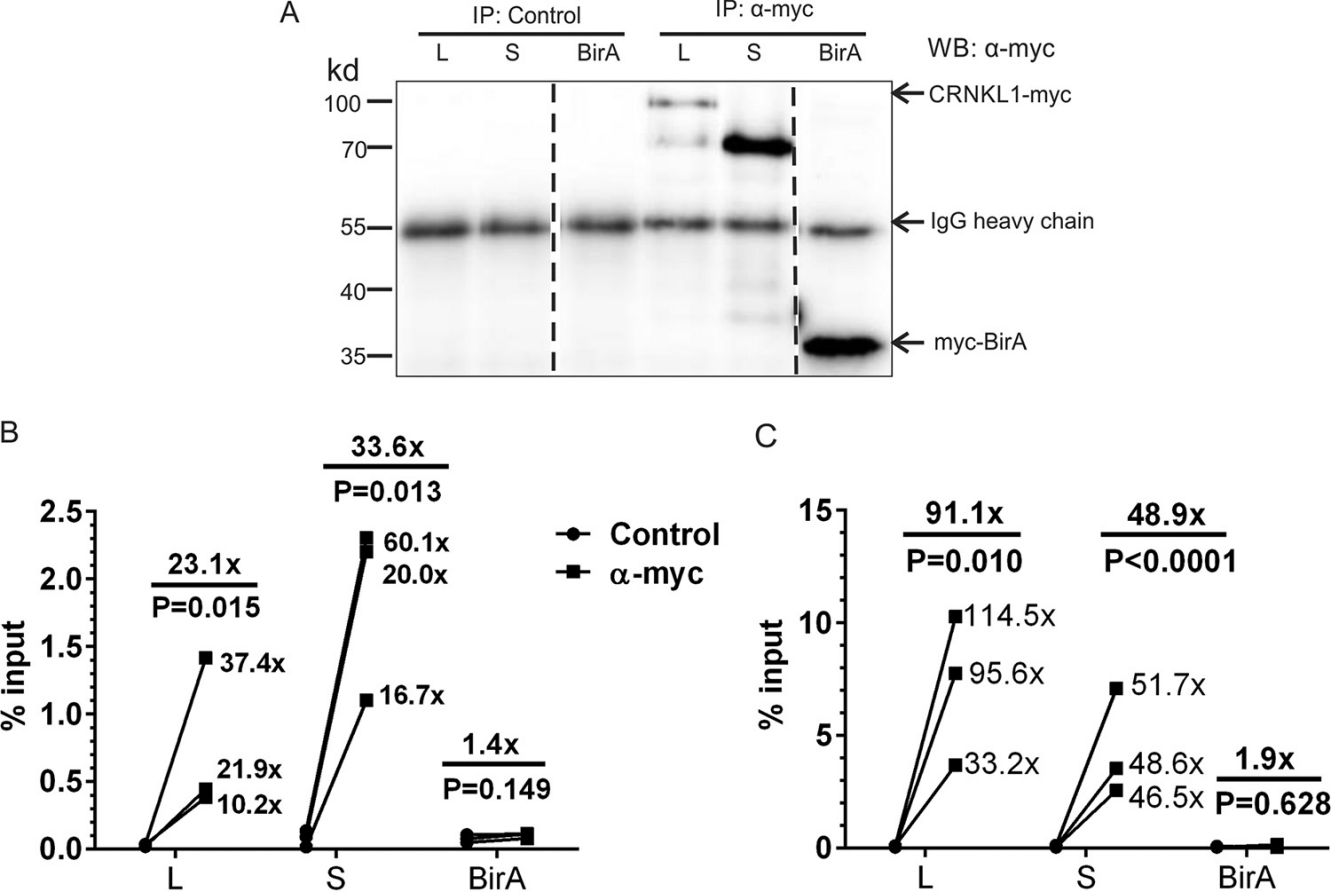
CRNKL1 proteins associate with HIV US RNA. (A) 293T cells transfected with expression plasmids encoding the myc-tagged CRNKL1 or an irrelevant control protein (BirA) were infected with VSV-G-pseudotyped HIV containing inactivating mutations in *rev* and *env* (HIV.r^−^e^−^). Nuclear fractions of these cells were immunoprecipitated with beads coated with anti-myc antibody or an isotype-matched control antibody (IgG2a). The precipitates were characterized by Western blot analysis with an anti-myc antibody. Dotted lines indicate excision of irrelevant lanes from the Western blot image. The precipitates described in panel A were also analyzed by RT-qPCR for HIV-US RNA and HIV-FS RNA levels. Results are expressed as percentages of HIV-1 US RNA (B) and HIV-1 FS RNA (C) copy numbers in the precipitate relative to the copy numbers in the nuclear fraction (input) prior to the immunoprecipitation. Fold changes of each replicate and mean fold changes are indicated. The ratio paired *t* test was used for statistical analysis. L, CRNKL1 large isoform; S, CRNKL1 small isoform.

As a nuclear retention factor of HIV-1 US RNA, CRNKL1 should either directly bind to the HIV-1 US RNA or be in the same RNP complex. Due to lack of a putative RNA binding motif, it was suggested that CRNKL1 binds to RNA via interactions with RNA binding proteins (65). However, CRNKL1 also contains a half-a-tetratricopeptide repeat (HAT) motif that has been implicated with direct RNA binding activity (66). To test whether CRNKL1 is indeed linked to nuclear HIV-1 US RNA, the two major isoforms of CRNKL1 (100 and 83 kDa [67]) were expressed in 293T cells with a myc tag. Pulldown experiments of nuclear extracts of these cells with an anti-myc antibody and an isotype-matched control antibody confirmed specific immunoprecipitation of both isoforms (Fig. 5A). Precipitates of the small and large isoforms of CRNKL1 also contained 34- and 23-fold higher levels of HIV-1 US RNA (Fig. 5B), confirming that at least 1% to 2% of the nuclear HIV-1 US RNA is associated with CRNKL1. No interaction was observed between HIV-1 US RNA and an irrelevant myc-tagged protein, BirA, revealing the specificity of HIV-1 US RNA interaction with CRNKL1. Comparable analyses for HIV FS RNA also revealed strong and specific associations with both isoforms of CRNKL1 (Fig. 5C). Since CRNKL1 is a member of the postcatalytic spliceosome (68), association of spliced transcripts with CRNKL1 complexes is expected. The different effects of the CRNKL1 knockdown on cytoplasmic RNA levels of HIV-1 US and FS RNA can therefore be best explained by the association of CRNKL1 with two or more RNP complexes differing in function.

### Knockdown of CRNKL1 enhances Gag expression in acutely infected 293T cells.

**FIG 6:**
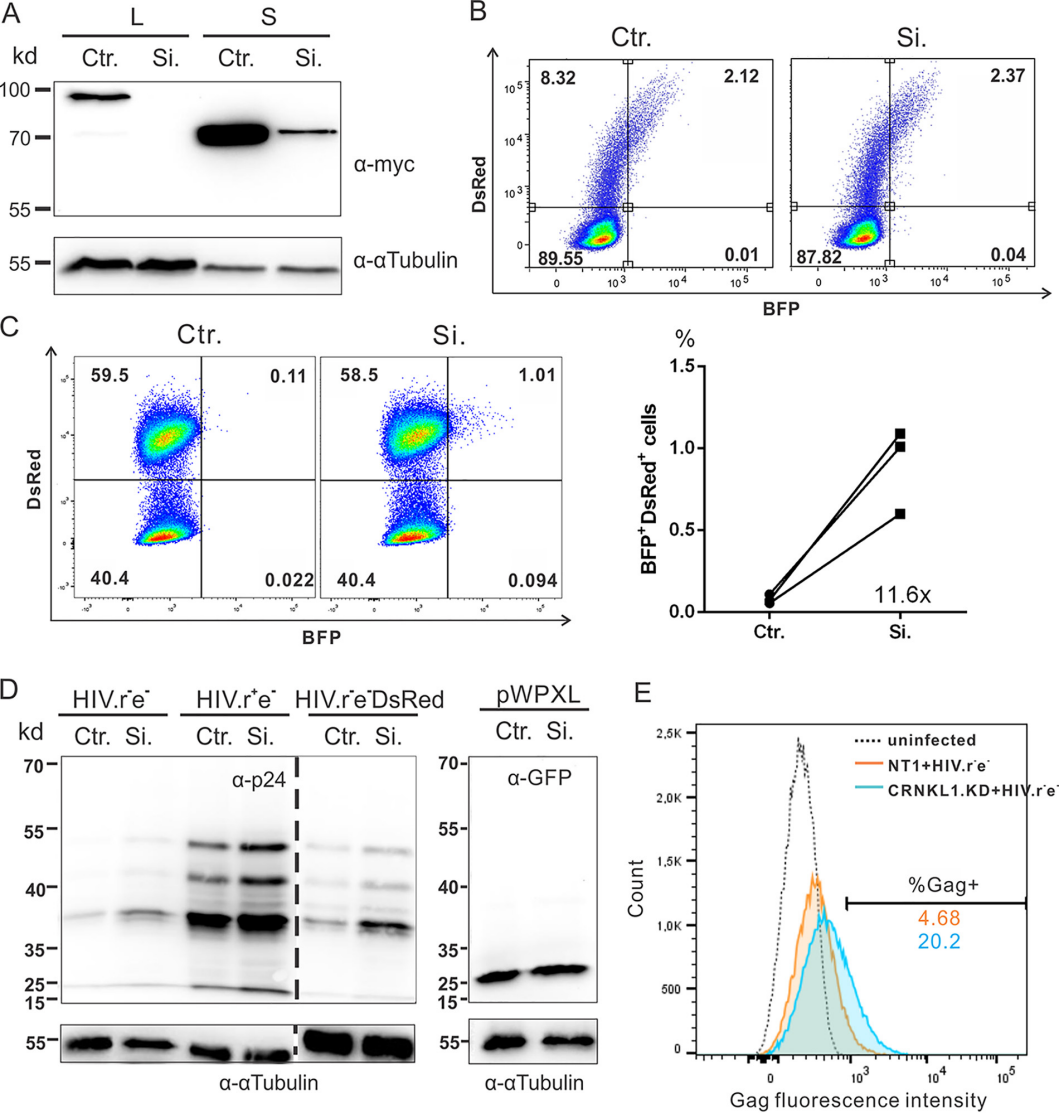
CRNKL1 knockdown increases expression of Gag after acute HIV infection. (A) Knockdown of CRNKL1 expression by siRNAs. CRNKL1-targeting siRNAs (Si.) and nontargeting control siRNAs (Ctr.) were cotransfected with myc-tagged CRNKL1 expression plasmids to test their efficacy and specificity. Exogenous CRNKL1 expression and endogenous α-tubulin expression in transfected 293T cells were monitored by anti-myc and anti-α-tubulin Western blot analyses, respectively. (B) Effect of CRNKL1 knockdown on Gag-BFP expression in transiently transfected cells. Targeting or nontargeting siRNAs were cotransfected with the HIV-dual-GT plasmid into 293T cells. Gag-BFP reporter expression was monitored by FACS analysis. (C) Effect of CRNKL1 knockdown on Gag-BFP expression in stably transduced cells. 293T cells stably transduced with HIV-dual-GT were transfected with the siRNAs and analyzed by flow cytometry. Shown are representative results from one experiment (left panel) and the percentage of BFP^+^ DsRed^+^ cells plotted from three independent experiments (right panel). (D) Effect of CRNKL1 knockdown on Gag expression in cells acutely infected with HIV-1. 293T cells were transfected twice within 4 days with siRNAs and then infected with VSV-G-pseudotyped HIV-1 *env* mutants with (HIV.r^−^e^−^) or without (HIV.R^+^e^−^) inactivating mutations of Rev. A reporter virus expressing wild-type Gag (HIV.r^−^e^−^DsRed) and a lentiviral vector expressing GFP from an internal promoter (pWPXL) were also included. Gag, GFP, and α-tubulin expression was analyzed by Western blotting using antibodies targeting the proteins indicated. The dotted line indicates excision of blank lanes from the Western blot image. (E) Effect of CRNKL1 knockdown on Gag expression in cells acutely infected with HIV-1 by flow cytometry. 293T cells were infected with CRNKL1 targeting or nontargeting CRISPR lentivirus and VSV-G-pseudotyped HIV.r^−^e^−^. Gag expression was analyzed on the single-cell level by intracellular staining for Gag and flow cytometry.

To exclude potential off-target effects of CRISPR/Cas-mediated genome editing, CRNKL1 expression levels were also reduced by small interfering RNAs (siRNAs). Cotransfection of 293T cells with a pool of four siRNAs targeting CRNKL1 and expression plasmids encoding the large and short forms of CRNKL1 led to strongly reduced CRNKL1 expression levels (Fig. 6A), confirming the functionality of the siRNAs. A simple model to test the effect of CRNKL1 knockdown on HIV-1 Gag expression might be to cotransfect these siRNAs targeting CRNKL1 with the HIV-dual-GT plasmid into 293T cells. However, by doing this, we could not observe a rise in the Gag-BFP reporter protein expression (Fig. 6B). In contrast, transfection of CRNKL1-targeting siRNAs into 293T cells stably transduced with the same HIV-dual-GT reporter virus revealed an 11.6-fold increase in the percentage of transduced cells expressing Gag-BFP (Fig. 6C). Given the complex and dynamic chromosomal architecture (69), the nuclear metabolism of mRNA expressed from a circular plasmid can differ from mRNA expressed from a proviral DNA integrated into a host chromosome. This may explain why the effect of CRNKL1 knockdown is detectable in transduced but not transfected cells.

To further explore the effect of the CRNKL1 knockdown in acutely infected cells in the presence and absence of Rev, 293T cells were first transfected with CRNKL1-targeting siRNAs or controls prior to infection with different VSV-G-pseudotyped HIV constructs. CRNKL1 knockdown enhanced Gag expression from HIV *env* mutants independent of Rev (Fig. 6D, left panel). This enhancement was not due to an increased susceptibility to virus infection after CRNKL1 siRNA treatment, since green fluorescent protein (GFP) expression levels after transduction with a lentiviral vector encoding the reporter gene from an internal promoter remained unchanged (Fig. 6D, right panel). The magnitude of the enhancing effect of CRNKL1 knockdown by Western blot analyses in infected 293T cells was lower than expected, based on the analyses of cytoplasmic RNA levels in transduced Jurkat cells enriched for CRNKL1 knockdown (Fig. 4B). As the enhancing effect of the CRNKL1 knockdown may be masked by background expression of Gag from cells expressing unreduced levels of CRNKL1, we also analyzed Gag expression after CRNKL1 knockdown on the single-cell level by flow cytometry. 293T cells were transduced with lentiviral vectors targeting CRNKL1 or a control vector and then infected with a VSV-G-pseudotyped HIV containing only inactivating mutations in *env* and *rev*. Intracellular staining for Gag revealed a 4-fold increase in the percentage of Gag-positive cells after CRNKL1 knockdown (Fig. 6E), indicating that the enhancing effect is not restricted to the HIV-dual-GT reporter virus or Jurkat cells.

### CRNKL1 knockdown shifts cytoplasmic levels in HIV-1 splice variants.

To confirm the role of CRNKL1 in the regulation of cytoplasmic HIV RNA levels and to explore potential effects of CRNKL1 on cytoplasmic levels of cellular RNAs, a transcriptomic analysis was performed after a short-term knockdown of CRNKL1. J-dual#3 reporter cells were again transduced with the CRNKL1 targeting vector, and Gag-BFP-positive cells were sorted by flow cytometry. RNA was extracted from the cytoplasmic fraction of sorted cells and J-dual#3 cells transduced with a control vector. The extracted RNAs from four biological replicates each were then enriched for mRNAs and sequenced by the Illumina HiSeq procedure. In addition to this, cytoplasmic mRNA was extracted from J-dual#3 cells expressing Gag-BFP after transduction with the Rev-encoding lentiviral vector and sequenced after mRNA enrichment.

To confirm the effectiveness of Cas9/sgRNA-mediated knockdown by transcriptomic analysis, a core target region in exon 3 of the CRNKL1 gene was defined as nucleotides (nt) −3 to +3 relative to the SpCas9 cleavage site. The number of transcriptome sequencing (RNA-seq) reads that contained 10 nucleotides upstream and downstream of the core target region were then determined (Fig. S5). While 55 reads on average mapped to the region flanking the core target region in the control cells, only a mean of 5.8 reads was detected in cells transduced with the CRNKL1 knockdown vector (Fig. S5). Larger deletions and substitutions outside the core target region probably reduce the number of reads that can be mapped to the flanking region. A comparison of the reads from CRNKL1 knockdown cells that can be mapped to the flanking region with the wild-type CRNKL1 core target region revealed insertions, deletions, and point mutations in 22 out of 23 reads. In control cells, only 2 of 219 reads contained single point mutations, clearly indicating efficient inhibition of CRNKL1 expression after Cas9/sgRNA targeting (Fig. S5).

10.1128/mBio.02525-20.5 FIG S5 Number of reads and deviations from the wild-type CRNKL1 target region after knockdown of CRNKL1. The CRNKL1 sgRNA target region with the predicted Cas9 cleavage site (arrow) and the PAM region (shaded in orange) are aligned with reads from the transcriptome from four control (NT1 exp1 to -4) and four CRNKL1 knockdown experiments (KD exp1 to -4). Underlined sequences were used to map reads to the target region. Point mutations, deletions (Δ), and insertions (Ι, with number of nucleotides inserted given above) are indicated. n, number of reads with the respective sequence; m, percent mutation rate calculated as [100 × (number of mutated reads/number of total reads mapped to the CRNKL1 target region)]. Download FIG S5, TIF file, 0.2 MB. Copyright © 2021 Xiao et al. 2021 Xiao et al. This content is distributed under the terms of the Creative Commons Attribution 4.0 International license.

**FIG 7:**
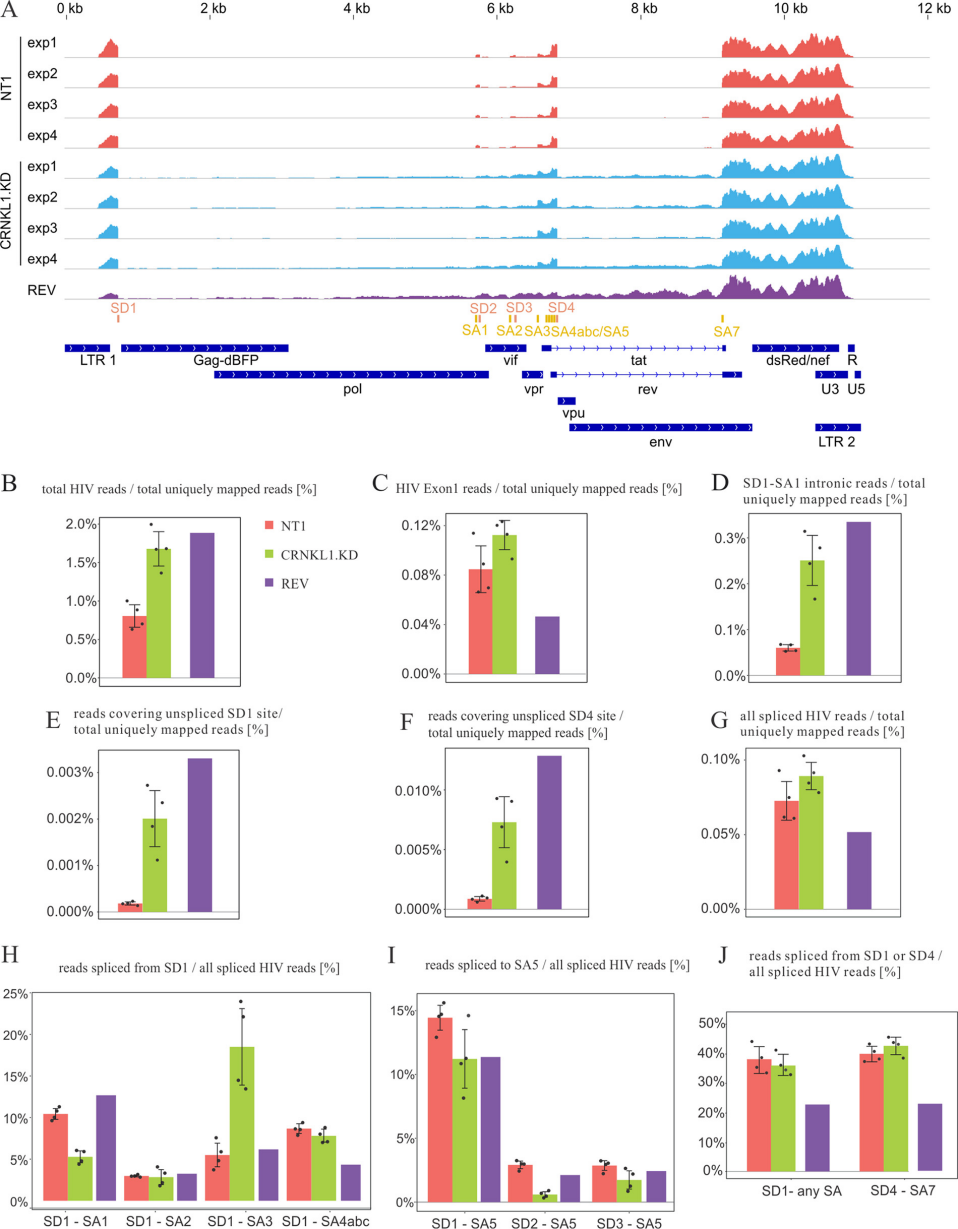
Effect of CRNKL1 depletion on HIV RNA splicing efficiency, accuracy, and export. (A) Alignments of reads from the transcriptomic analysis to HIV-dual-GT. Shown are coverage profiles for the following individual RNA-seq samples, indicated by color (from top to bottom): the nontargeting control (NT1, red), CRNKL1 knockdown (cyan), and *rev* overexpression (purple). Splice sites are shown in orange (donors) and dark yellow (acceptors). Coding regions and long terminal repeats (LTRs) are indicated in dark blue. (B) Percentage of all HIV reads among the total (HIV plus human) uniquely mapped reads. (C) Percentage of reads mapping to HIV exon 1 (from the transcription start site to SD1) among the total uniquely mapped reads. (D) Percentage of reads in the first intron between SD1 and SA1 among the total uniquely mapped reads. (E) Percentage of reads across the unspliced SD1 site among the total uniquely mapped reads. (F) Percentage of reads across the unspliced SD4 site among the total uniquely mapped reads. (G) Percentage of reads spanning SD and SA sites (spliced reads) among the total uniquely mapped reads. (H) Percentages of reads spanning SD1 and the indicated splice acceptor sites among all spliced HIV reads. For SD1-SA4abc, reads spliced from SD1 to SA4a, SA4b, and SA4c are summed up. (I) Percentages of reads spanning the indicated splice donor sites and SA5 among all spliced HIV reads. (J) Percentage of reads spliced from SD1 to any SA site or from SD4 to SA7 among all spliced HIV reads. (B to J) Shown are averages and standard deviations for the control and CRNKL1 knockdown samples. Individual values are shown as black dots. For the *rev* overexpression sample (purple), the individual value is shown.

HIV reads were mapped to the proviral DNA of HIV-dual-GT, revealing more reads mapping to intronic regions after CRNKL1 knockdown and Rev expression than in the controls (Fig. 7A). The percentage of reads mapping to HIV among all the reads uniquely mapping to either the human or the viral genome (total uniquely mapped reads) was enhanced 2-fold in CRNKL1 knockdown cells (Fig. 7B). This was not due to an enhanced transcriptional activity, since the percentage of reads mapping to exon 1, which is shared by all HIV transcripts, was affected only marginally (Fig. 7C). Changes in HIV-1 US RNA were assessed by determining the percentage of reads mapping to the first intron of HIV-1 between splice donor 1 (SD1) and splice acceptor 1 (SA1) (Fig. 7D) or mapping to the unspliced SD1 site (Fig. 7E), revealing 4- and 12-fold increases under CRNKL1 knockdown conditions, respectively. The percentage of reads mapping to the unspliced SD4 site and therefore containing the *env* intron also increased by a factor of 9 (Fig. 7F). The change in the percentage of all spliced HIV reads was negligible (Fig. 7G), indicating that knockdown of CRNKL1 does not reduce splicing of HIV RNAs in general.

To further explore differences in cytoplasmic levels of differentially spliced HIV-1 transcripts, the precise splice site usage was also systematically analyzed by calculating the percentage of reads covering specific SD-SA pairs among all spliced HIV reads (Fig. S6A and B). This revealed that knockdown of CRNKL1 reduced cytoplasmic HIV transcripts spliced from SD1 to SA1 approximately 2-fold, while transcripts spliced from SD1 to SA3 were enhanced 3-fold (Fig. 7H). To further assess the effect of the CRNKL1 knockdown on the major Env-encoding transcript, the percentage of reads spliced to SA5 was determined (Fig. 7I). Independent of the SD sites, usage of SA5 tended to be reduced, which is similar to an effect recently reported for the splicing factor DDX17 (70). Moreover, the level of fully spliced transcripts seemed unaffected by the CRNKL1 knockdown, since the percentages of reads spliced either from SD1 to any splice acceptor site or from SD4 to SA7 were nearly the same (Fig. 7J).

10.1128/mBio.02525-20.6 FIG S6 Changes in splice site usage of HIV RNAs upon CRNKL1 depletion. (A) Heat map representing, for every row, the relative frequency of indicated HIV-1 splice donor-splice acceptor pairs. Colors represent the log_10_-transformed percentages relative to all spliced HIV reads, as shown in the color bar below the heat map. Rows are sorted according to the splice donor sites. (B) Same as described for panel A but sorted for splice acceptor sites. Download FIG S6, TIF file, 0.4 MB. Copyright © 2021 Xiao et al. 2021 Xiao et al. This content is distributed under the terms of the Creative Commons Attribution 4.0 International license.

### CRNKL1 is a regulator of cytoplasmic cellular mRNA levels.

**FIG 8:**
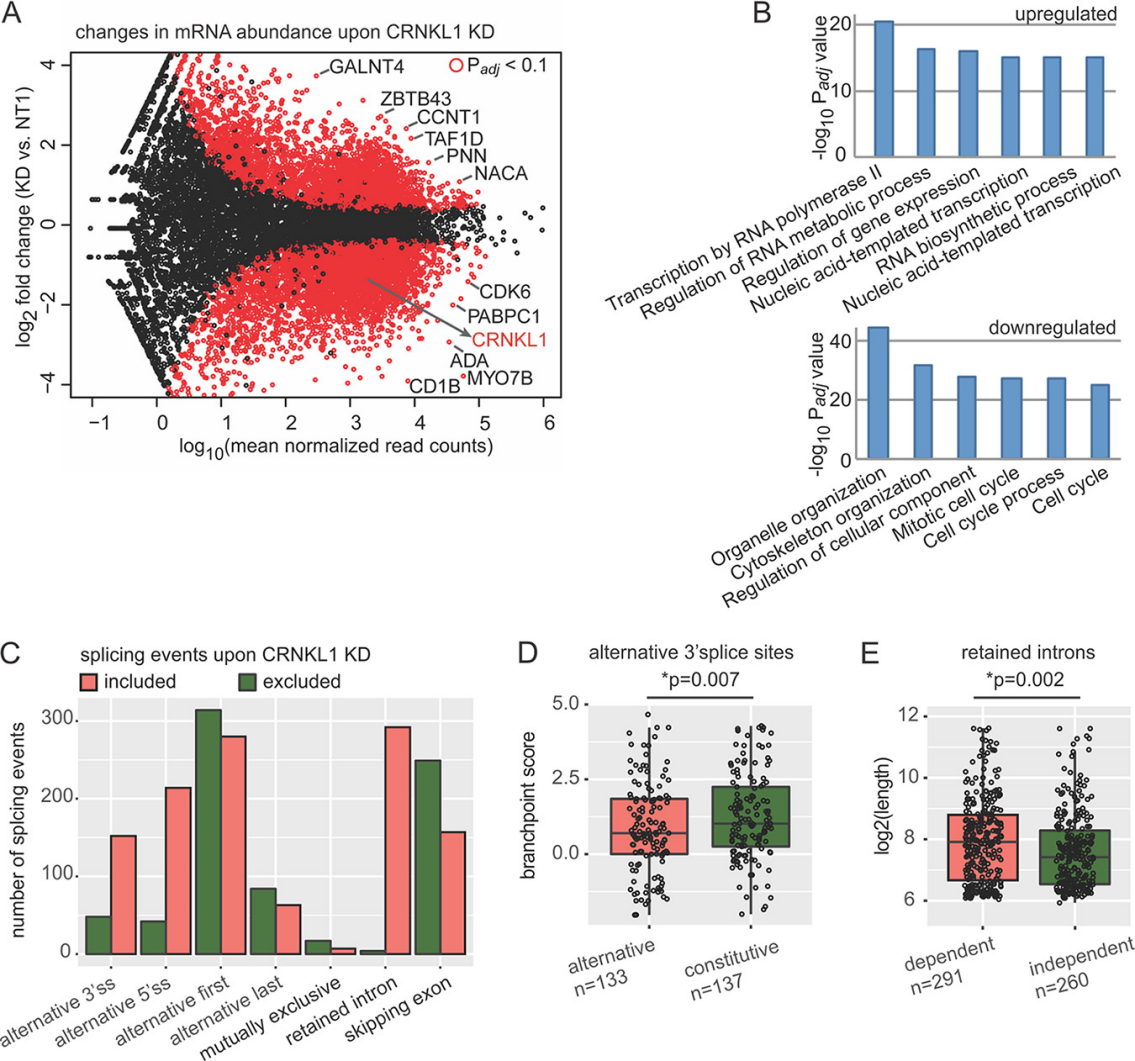
Changes in the host transcriptome upon CRNKL1 knockdown (KD). (A) RNA-seq differential expression analysis of mRNA abundance upon CRNKL1 KD. An MA plot shows differences in the abundance of protein coding transcripts in CRNKL1 KD versus control (NT1) conditions. mRNAs with significant differences are depicted in red (*P*_adj_ < 0.1). (B) Gene Ontology enrichment analysis of mRNAs with upregulated and downregulated expression upon CRNKL1 KD. The top enriched GO Biological Process terms are shown. (C) Upregulated (included) (ΔPSI [KD − NT1] > 0.25, *P*_adj_ < 0.01) and downregulated (excluded) (ΔPSI [KD − NT1] < −0.25, *P*_adj_ < 0.01) splicing events were quantified from the RNA-seq CRNKL1 KD experiment. The bar plot shows absolute numbers of splicing events that are up- or downregulated upon CRNKL1 knockdown. (D) Branch point scores were calculated by BPfinder, and alternative 3′ splice sites with higher usage in CRNKL1 KD condition were compared to 3′ splice sites of the same genes with unchanged (constitutive) usage. Box plots and data points are shown. The Wilcoxon rank sum test was used to test for significance. Absolute numbers of 3′ splice sites included in the analysis for each group are indicated. (E) Length of retained introns with higher inclusion upon KD (CRNKL1 dependent) was compared to the length of unchanged introns upon KD (CRNKL1 independent), which were located in the same transcripts as CRNKL1-dependent ones. Box plots and data points are shown. The Wilcoxon rank sum test was used to test for significance. Absolute numbers of retained introns analyzed in both groups are indicated. PSI, percent spliced-in for splicing events.

The influence of the CRNKL1 knockdown on cytoplasmic mRNA levels was also determined (Fig. S7A and B). To display the results, the log_2_-transformed expression differences were plotted against the expression level for each gene (Fig. 8A). To reduce noise and arbitrarily large fold changes from low-expression genes, the log_2_ fold changes are shrunk, putting less weight on low-expression genes. Significant differences after correction for multiple testing revealed the upregulation of 1,249 and downregulation of 2,569 mRNAs, respectively. Two hundred ten mRNAs even showed a more than 4-fold (log_2_ fold change, >2) increase after knockdown of CRNKL1. Interestingly, upregulated mRNAs were enriched for the functional categories involved in transcription (Fig. 8B) while downregulated mRNAs were more frequently associated with functions in cell cycle and organelle organization (Fig. 8B).

10.1128/mBio.02525-20.7 FIG S7 Quality control analyses for the transcriptomic data and additional analyses on differential splicing and intronic features. (A) Principal component analysis (PCA) of exonic read counts. Variance was assessed between biological replicates (1 to 4) and conditions (NT1, KD, REV). (B) Pairwise assessment of Pearson correlation coefficients between all exonic read counts from all RNA-seq samples. (C) Differential analysis of splicing events upon CRNKL1 KD. Volcano plot of ΔPSI (KD − NT1) values versus negative log10-transformed *P*_adj_ values is shown. To select up- and downregulated splicing events upon CRNKL1 KD (blue and red data points), the following cutoffs were used, respectively: ΔPSI (KD − NT1) > 0.25, *P*_adj_ < 0.01; and ΔPSI (KD − NT1) < −0.25, *P*_adj_ < 0.01. (D) Pairwise assessment of Pearson correlation coefficients between all splicing event PSI values from all RNA-seq samples. (E) The lengths of polypyrimidine tracts of alternative 3′ splice sites with higher usage under the CRNKL1 KD condition were compared to those of constitutive 3′ splice sites present in the same mRNAs. The Wilcoxon rank sum test was used to test for significance. Absolute numbers of 3′ splice sites in both groups are indicated. (F) Polypyrimidine track length was obtained by BPfinder for retained introns with higher inclusion upon KD (CRNKL1 dependent) and compared to that of unchanged retained introns upon KD (CRNKL1 independent), which were located in the same transcripts as CRNKL1-dependent ones. Box plots and data points are shown. The Wilcoxon rank sum test was used to test for significance. Absolute numbers of retained introns in both groups are indicated. (G) The GC content of retained introns with higher inclusion upon KD (CRNKL1 dependent) was compared to the GC content of unchanged introns upon KD (CRNKL1 independent), which were located in the same transcripts as CRNKL1-dependent ones. Box plots and data points are shown. The Wilcoxon rank sum test was used to test for significance. Absolute numbers of retained introns in both groups are indicated. Download FIG S7, TIF file, 0.8 MB. Copyright © 2021 Xiao et al. 2021 Xiao et al. This content is distributed under the terms of the Creative Commons Attribution 4.0 International license.

Given the close link between RNA export and splicing, a more general role of CRNKL1 in cytoplasmic levels of splice variants was analyzed upon CRNKL1 knockdown. Individual splicing events (including skipped exons, alternative splice sites, and retained introns) were quantified using the percent spliced-in (PSI) metric (97), and differential analysis was carried out by SUPPA (71). Of all quantified splicing events in cytoplasmic mRNAs upon CRNKL1 depletion, 2.4% were upregulated and 1.6% were downregulated (Fig. S7C and D). Similarly, alternative 3′ and 5′ splice sites were more frequently detected upon CRNKL1 knockdown, suggesting that CRNKL1 inhibits usage of alternative splice sites (Fig. 8C).

A total of 295 introns were more frequently retained in cytoplasmic mRNAs after CRNKL1 knockdown (Fig. 8C; Table S2). Further analyses revealed that not all introns of the same transcript were affected in the same way (Fig. S8) but rather that intron retention was highly specific for one or a few introns of the same gene. Enhanced cytoplasmic levels of intron-retaining mRNAs could be confirmed by real-time PCR using total cytoplasmic RNAs extracted for RNA-seq and primers spanning the respective exon-intron junctions (Fig. S8).

10.1128/mBio.02525-20.8 FIG S8 Examples and validation of intron retention in cellular transcripts after CRNKL1 knockdown. Mapping of the reads to genes with enhanced intron retention (RPL10, C19orf53, CD320), an alternative 5′and 3′ splice site usage (5th intron of AURKB), and an unaffected gene (EIF6) are shown for the four replicates of control (NT1) and CRNKL1 knockdown cells and the *rev*-expressing cells. For RPL10 and C19orf53, intron retention was confirmed by RT-qPCR. Binding sites for specific primers spanning the exon-intron junctions are indicated by green arrows. RT-qPCRs were performed for relative quantification of respective intron-retaining transcripts using the cytoplasmic RNAs from the RNA-seq experiment. Δ*C_T_* (NT1 − KD) ± SEM values obtained from the four replicates are indicated. Download FIG S8, TIF file, 0.7 MB. Copyright © 2021 Xiao et al. 2021 Xiao et al. This content is distributed under the terms of the Creative Commons Attribution 4.0 International license.

10.1128/mBio.02525-20.10 TABLE S2 Splicing events increased upon CRNKL1 knockdown. All splicing events with a ΔPSI (KD − NT1) of >0.25 and a *P*_adj_ of <0.01 are listed. Download Table S2, XLSX file, 0.2 MB. Copyright © 2021 Xiao et al. 2021 Xiao et al. This content is distributed under the terms of the Creative Commons Attribution 4.0 International license.

Genes containing introns or alternative splice sites that were upregulated after CRNKL1 knockdown were not enriched for particular functional categories (data not shown). Enhanced detection of introns or alternative splice sites in cytoplasmic mRNAs after CRNKL1 knockdown indicate that CRNKL1 suppresses either these splicing events or the nuclear export of the respective splice variants. To gain insight into the principles of regulation of these CRNKL1 knockdown-dependent introns and alternative splice sites, we analyzed common features of these splicing events, including splice site strength, branch point and polypyrimidine tract score, GC content, and intron length. CRNKL1 knockdown-dependent alternative 3′ splice sites showed significantly lower branch point scores than constitutive 3′ splice sites (Fig. 8D) but a similar length of polypyrimidine tracts (Fig. S7E). We also found that CRNKL1-dependent introns were significantly longer than CRNKL1-independent introns within the same transcript (Fig. 8E) and tended to have longer polypyrimidine tracts (Fig. S7F) but showed no difference in GC content (Fig. S7G).

## DISCUSSION

Using a genome-wide screen for cellular factors repressing expression of an HIV-1 structural protein from the unspliced RNA, we identified 10 target proteins linked to mRNA metabolism, with nine of them associated with the spliceosome. Interestingly, five of them (ISY1, BUD31, XAB2, CRNKL1, and CWC22) are found in the Prp19-associated complex. This complex is important for stabilizing the spliceosome, which is also involved in DNA repair, transcriptional elongation, and RNA export (57). Given that the yeast homolog of WDR70 was reported to interact with Prp19 (72) and is now shown to affect RNA splicing, it could be a new member of the Prp19-associated complex. Due to the close linkage of splicing with RNA stability and nuclear RNA export, it is difficult to dissect the precise mechanism by which deletion of the identified nuclear proteins enhance cytoplasmic RNA levels of HIV-1 US RNA.

**FIG 9:**
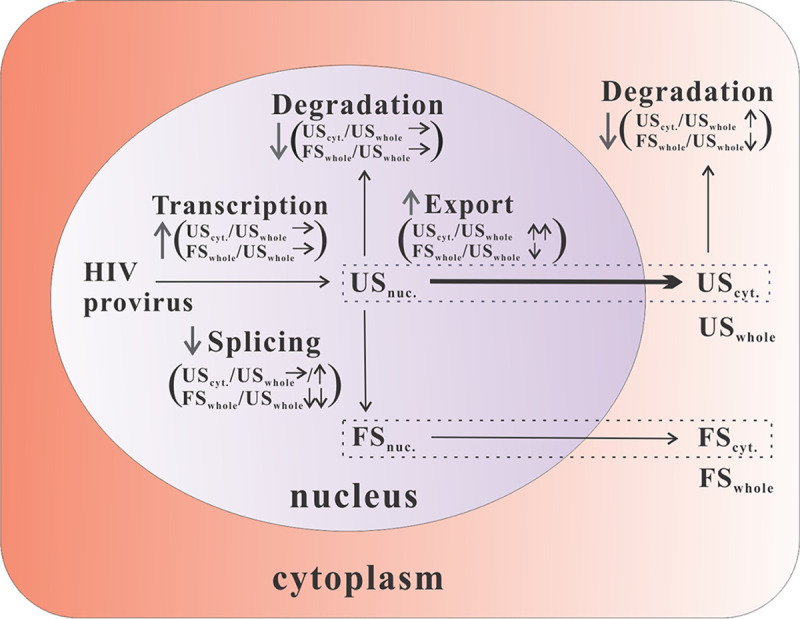
Potential mechanisms leading to increased cytoplasmic levels of unspliced HIV-1 RNA. Graphical representation of HIV-1 RNA metabolism and expected consequences of upregulation (↑) or downregulation (↓) regulation or no change (→) of the respective steps on the nuclear export efficacy (US_cyt._/US_whole_) and splicing efficacy (FS_whole_/US_whole_). The nuclear export pathway is highlighted in bold.

Theoretically, enhanced transcription, reduced nuclear or cytoplasmic degradation, decreased splicing, and/or enhanced nuclear export can increase cytoplasmic levels of HIV-1 US RNA (US_cyt_) (Fig. 9). Enhanced transcription and decreased nuclear degradation seem unlikely, since the percentage of reads mapping to the first HIV-1 exon present on all viral transcripts was not significantly enhanced (Fig. 7C). The absence of enhanced cytoplasmic levels of FS RNAs and DsRed expression levels from the dual reporter construct (Fig. 4B; Fig. S2A) also argues against such a mechanism. Assuming a single pool of nuclear HIV-1 US RNA that could follow either of the two remaining pathways, differential effects on the nuclear export efficiency (US_cyt._/US_whole_) and splicing efficacy (FS_whole_/US_whole_) are expected (Fig. 9). Enhancing nuclear export of HIV-1 US RNA should strongly enhance the US_cyt._/US_whole_ ratio. Since rapid export may reduce splicing and degradation, the FS_whole_/US_whole_ ratio may decrease responsively, although to a lower extent. Reduced splicing should primarily decrease the FS_whole_/US_whole_ ratio but not the US_cyt._/US_whole_ ratio (Fig. 9). Similar to an enhanced nuclear export, reduced degradation of cytoplasmic HIV-1 US RNA should also lead to enhanced US_cyt._/US_whole_ and decreased FS_whole_/US_whole_ ratios (Fig. 9), but the association of the identified proteins with the spliceosome localized in the nucleus argues against such a cytoplasmic effector mechanism.

Clearly, ablation of all six target genes tested reduced the splicing efficacy (FS_whole_/US_whole_), indicating that the targeted genes are enrolled in splicing and/or nuclear retention. The relative contribution of reduced splicing versus enhanced nuclear export of the HIV-1 US RNA differed between the different target genes. The XAB2 knockdown decreased the splicing efficacy 12.5-fold and the nuclear export efficacy of the HIV-1 US RNA 11.7-fold. Since splicing and nuclear export efficacies were affected to a similar degree, it is not possible to conclude whether XAB2 acts predominantly as a splicing or a nuclear retention factor. This is consistent with previous observations that depletion of splicing factors (e.g., SF1, U2AF65, and Prp19) can cause pre-mRNA leakage into the cytoplasm (36, 73–75). In contrast to the effect of the XAB2 knockdown, the splicing efficacy after knockdown of CRNKL1 is decreased only 4.0-fold, while the nuclear export efficiency based on the HIV-1 US RNA increases 34.6-fold. Since the ratio of HIV-1 FS to US transcripts in the cytoplasmic RNA is also reduced to a larger extent than in the whole-cell RNA, enhanced nuclear export of the HIV US RNA after inactivation of the target gene seems to play a dominant role. This indicates that CRNKL1 is required primarily for retention of the HIV-1 US RNA in the nucleus and not for its splicing. The precise molecular mechanism by which the CRNKL1 knockdown leads to a more than 100-fold enhancement of cytoplasmic HIV US RNA levels remains to be further investigated. Our observation that targeting of CRNKL1 did not enhance cytoplasmic levels of HIV-1 FS RNA, which uses the default TAP/NXF1 export pathway, indicates that CRNKL1 is not a global nuclear mRNA retention factor. Our transcriptomic analysis also indicates a highly selective differential regulation of cytoplasmic levels of HIV-1 splice variants. The only spliced HIV-1 transcripts that were clearly upregulated by CRNKL1 knockdown have spliced SD1 to the SA3 site (Fig. 7G) and may encode Tat. Whether this represents an upregulation of *env* intron-retaining transcripts or the Tat-encoding fully spliced transcript is unclear. The enhanced percentage of cytoplasmic HIV transcripts harboring an unspliced SD4 site (Fig. 7F) and the unchanged percentage of transcripts harboring a spliced SD4 site (Fig. 7J) argue for the former.

The transcriptomic analyses after CRNKL1 knockdown also revealed a selective regulation of cytoplasmic levels of a subset of cellular mRNAs. We found that 11.0% and 20.1% of expressed mRNAs in the cytoplasm were up- and downregulated, respectively. Functionally, the upregulated mRNAs are associated with transcription, while downregulated mRNAs are more frequently associated with the cell cycle and organelle organization, suggesting that CRNKL1 participates as a master regulator in these cellular programs. The large percentage of cellular genes affected by CRNKL1 knockdown may be due not only to a direct consequence of reduced CRNKL1 levels but also to secondary effects requiring further investigations. Depletion of an essential gene such as the CRNKL1 gene may induce cell death. However, at the time of the analyses, the pattern of forward and side scatters during flow cytometric analysis of BFP-positive CRNKl1 knockout cells and BFP-negative cells were indistinguishable, while dead cells clearly differed (data not shown). Consistently, the percentage of BFP-positive cells remained unchanged for several days after the day of the analyses. In addition, the transcriptomic pathway analyses did not provide any evidence for upregulation of stress response genes or apoptosis-related genes.

Since altered nuclear export after CRNKL1 depletion could result in differences in the abundance of different cytoplasmic mRNA isoforms, we investigated alternatively spliced cytoplasmic isoforms upon CRNKL1 knockdown. Upon CRNKL1 depletion, a much higher number of included introns and alternative 3′ and 5′ splice sites than the excluded ones were detected, suggesting that CRNKL1 promotes splicing of these events or highly selective nuclear retention. Intron retention in the absence of CRNKL1 was highly restricted and even varied among introns of the same gene (see Fig. S8 in the supplemental material). Searching for common properties of introns that were retained revealed that their mean length was significantly greater than that of introns with unchanged inclusion upon CRNKL1 depletion. In addition, usage of 3′ splice sites with lower branch point scores seems to be blocked in the presence of CRNKL1. Therefore, the sequence features of CRNKL1-dependent splicing events seem to reside at the 3′ end rather than at the 5′ end of the intron.

As a component of the core splicesome, CRNKL1 is well conserved from yeast to human, is ubiquitously expressed in various tissues, and has been shown to affect splicing reactions *in vitro* (53, 54, 67). Since CRNKL1 has a HAT motif composed of alpha-helix pair repeats and belongs to the Prp19-associated complex, it has been implicated primarily in DNA repair and cell cycle regulation (76, 77). Noticeably, this motif may also confer direct RNA-binding activity. Beyond that, CRNKL1 has been reported to be involved in RNA elongation and RNA export (78, 79). The role of CRNKL1 in RNA extension and export is especially interesting, demonstrating that CRNKL1 has interactions with the TREX exporting machinery at an early stage of RNA synthesis, which might determine the “export” or “retain” status of an RNA.

Mechanistically, one plausible hypothesis based on the nuclear retention observed for the HIV-1 unspliced RNA is that a nuclear RNP complex containing CRNKL1 binds to RNA motifs present in a selective subset of cellular and viral introns, leading to their nuclear retention and splicing. In support of this hypothesis, we observed that CRNKL1 is associated with nuclear HIV-1 unspliced RNA (Fig. 5C). The precise binding motif that is bound by CRNKL1 or CRNKL1-containing RNP complexes needs to be further investigated. Binding of CRNKL1-containing RNPs to binding motifs on cellular transcripts leading to nuclear retention and degradation may also explain the increase in cytoplasmic levels of cellular mRNAs upon CRNKL1 knockdown. However, it is not possible to exclude that changes in cellular mRNA levels are secondary effects of the CRNKL1 knockdown.

In summary, our study identifies three spliceosomal proteins that are required for nuclear retention of the HIV-1 unspliced RNA and indicates that CRNKL1-dependent nuclear retention is a novel mechanism for the regulation of cytoplasmic levels of intron-retaining HIV-1 mRNAs that HIV-1 may have hijacked to regulate its complex splicing pattern.

## MATERIALS AND METHODS

### Plasmids.

Plasmids expressing viral proteins, HIV-1 Rev (pcRev), HIV-1 Tat (pcTat), HIV-1 codon-optimized Gag-Pol (Hgp^syn^), and VSV-G (pHiT-G), were described previously (27, 62). Lentiviral packaging plasmid pPAX2 and pMD2.G were obtained from Addgene (no. 12260 and 12259). HIV-1 proviral plasmid HIV.R^+^e^−^ is based on the HIV-1 molecular clone NL4-3 and contains a 4-nucleotide deletion at its NheI site, leading to inactivation of *env*. The plasmid HIV.r^−^e^−^ contains an additional mutation of the start codon of *rev* and a premature stop codon in the first *rev* exon (63). The plasmid HIV.r^−^e^−^ DsRed contains the mutations that HIV.r^−^e^−^ has and is further mutated with *nef* by replacing it with DsRed as described for HIV-dual-GT below. Lentiviral vector pWPXL delivering a GFP gene driven by an internal EF1α promoter was obtained from Addgene (no. 12257). The Rev coding lentivector pWPXL-Rev was cloned by replacing the GFP sequence between the MluI and EcoRI site in the pWPXL vector with the Rev coding sequence from pcRev. The large isoform of myc-tagged CRNKL1 coding plasmid CRNKL1(L)-myc was obtained from Origene (no. RC220334). The coding sequence of large CRNKL1 relative to amino acids (aa) 1 to 169 was deleted from CRNKL1(L)-myc, generating the expression construct for small isoform CRNKL1 [CRNKL1(S)-myc].

Proviral reporter plasmid HIV-dual-GT was constructed based on HIV.r^−^e^−^. A cassette containing mTagBFP2, a PEST coding sequence (destabilized BFP), and two stop codons were ligated in frame to the 3′ end of *gag*, forming a new ORF for Gag-BFP. The slippery sequence TTTTTTA (nt 2085 to nt 2091 of GenBank entry AF324493.2) was mutated to CTTCCTG to prevent Gag-Pol translation (80). The coding sequence of Nef close to its 5´end (nt 8796 to nt 8880 of GenBank entry AF324493.2) was replaced by a cassette consisting of a 3×Ala linker sequence and the DsRed max coding region followed by two stop codons. The sequence (nt 5131 to nt 6297 of GenBank entry AF324493.2) ranging from *vif* to *vpu* was replaced by the corresponding region of NL4-3-Δ6-drEGFP (81) harboring inactivating point mutations in *vif*, *vpr*, and *vpu*. The *rev* ORF was inactivated as described above for HIV.r^−^e^−^ by PCR mutagenesis. The plasmid HIV-dual-GT including the final sequence is available from AddGene (plasmid no. 122696). Individual CRISPR knockout plasmids were constructed based on lentiCRISPRv2 (no. 52961; Addgene) and GeCKO v2 sgRNA target sequences. All sgRNA sequences of the GeCKO v2 library can be found at www.addgene.org/pooled-library/zhang-human-gecko-v2/. The different sgRNA sequences selected for validation are provided in Table S1A in the supplemental material. Each of the sgRNA sequences was cloned into the vector by following the instructions on the Addgene webpage (www.addgene.org/52961/).

### GeCKO v2 library.

The pooled human GeCKO v2 CRISPR knockout plasmid library was a gift from Feng Zhang (47) and was obtained from Addgene (no. 1000000048 and 1000000049). It is supplied as two sublibraries, each containing three different sgRNA constructs targeting each gene in the genome. Amplification of the lentiviral vector plasmid library was performed essentially as described by the contributors’ protocol (47). The complexity of the library was confirmed by HiSeq NGS of the pooled plasmid library (data not shown).

### Cell culture.

HEK293T (DSMZ) and TZM-bl (82) (NIH AIDS reagent program) cell lines were maintained in Dulbecco's Modified Eagle's medium (DMEM) supplemented with 10% fetal calf serum (FCS) and 1% penicillin/streptomycin (D10). Jurkat, clone E61 (ATCC), and reporter cell lines J-dual#3 and J-dual#6 were maintained in Roswell Park Memorial Institute (RPMI) 1640 supplemented with 10% FCS and 1% penicillin/streptomycin (R10). After expansion, J-dual#3 and J-dual#6 stocks were stored frozen in aliquots at −80°C. A fresh aliquot was thawed and expanded in each assay.

### Cell transfection.

HEK293T cells were routinely transfected by the polyethylenimine (PEI) precipitation method (62). In general, 3.5 million cells per T25 flask (CellStar) were seeded 24 h before transfection. For each transfection, plasmid DNA was mixed with calf thymus carrier DNA (Thermo Fisher Scientific) to give a total of 10 μg DNA. The 10 μg DNA was mixed with 15 µl PEI (1 µg/µl; Sigma-Aldrich) and added to the cells. After 8 h of incubation with the cells, the transfection medium was replaced with fresh D10 medium. Supernatants or cells were harvested 48 h posttransfection. To produce pseudotyped HIV.R^+^e^−^, HIV.r^−^e^−^, HIV.r^−^e^−^DsRed, and HIV-dual-GT vector particles, 3 μg proviral plasmid, 3 μg Hgp^syn^, 2 μg pHiT-G, 1 μg pcTat, and 1 μg pcRev were used. To produce individual CRISPR knockout (CRISPR-KO) lentiviral vector particles, 5 μg lentiCRISPRv2 plasmid, 3.75 μg pPAX2, and 1.25 μg pMD2.G were used. To produce the pooled human GeCKO v2 lentiviral vector library, 24 million 293T cells were seeded into a T175 tissue culture flask. At 24 h later, 20 μg GeCKO v2 library plasmids, 15 μg pPAX2, 5 μg pMD2.G, and 120 μl GenJet transfection reagent (SignaGen) were used for transfection. HIV-dual-GT-transduced Jurkat cells were transfected by the electroporation method described elsewhere (83). In brief, 5.0 million stably transduced cells were electroporated with 50 μg plasmids by the Gene Pulser Xcell electroporation system (Bio-Rad) at 250 V and 1,500 μF. At 48 h posttransfection, cells were harvested for flow cytometry analysis. The transfection efficiency after expression of a GFP reporter plasmid was estimated to be between 10% and 20%.

### siRNA transfection.

A pool of four siRNA duplexes targeting CRNKL1 (Dharmacon; sequences shown in Table S1E) or the control, siGENOME nontargeting siRNA control pool no. 2 (D-001206-14-50; Dharmacon), was transfected into 293T or 293T reporter cells using the DharmaFECT 1 transfection reagent (Dharmacon). For 2 × 10^5^ cells, 10 pmol siRNA with or without 100 ng plasmid was transfected essentially as described by the manufacturer. CRNKL1-myc expression by siRNA knockdown was examined by Western blotting at 2 days posttransfection; Gag-BFP expression influenced by CRNKL1 knockdown was measured by fluorescence-activated cell sorting (FACS) at 4 days posttransfection. To enhance the CRNKL1 knockdown efficiency, 4 × 10^5^ cells were given two times 60 pmol siRNAs in 4 days and then infected with VSV-G-pseudotyped HIV variants or a control lentivector at an MOI of 1. At 2 days postinfection, the cells were lysed using lysis buffer, whose volume was adjusted by cell number.

### Western blot analyses.

Immunoblotting for HIV-1 Gag/CA p24 was described previously (63). Immunoblotting for Rev was performed with primary antibody sheep anti-Rev (1:4,500, no. H6006; US Biological Life Sciences) and secondary antibody rabbit anti-goat Ig/HRP (1:5,000, no. P0160; Dako). Immunoblotting for myc-tagged proteins and for GFP was performed using primary antibodies monoclonal mouse anti-myc tag (1:8,000, no. 2276; Cell Signaling Technology) and monoclonal mouse anti-GFP (1:500, no. sc-9996; Santa Cruz) in combination with the secondary antibody goat anti-mouse IgG (1:5,000, no. 115-035-146; Dianova). Immunoblotting for α-tubulin was performed using primary antibody polyclonal rabbit anti-α-tubulin antiserum (1:4,000, no. 600-401-880; Rockland), and secondary swine anti-rabbit Ig/horseradish peroxidase (HRP) (1:5,000, no. P0217; Dako). In experiments shown in Fig. 1B, 6A, and 6C, anti-α-tubulin blotting was conducted after stripping the blotted anti-CA and anti-myc membranes with stripping buffer (no. 2504; Millipore). The membranes were then reblocked and subsequently reblotted with antibodies as described above. Immunoblotting for LaminB was performed with primary antibody goat anti-LaminB (C-20) (1:500, no. sc-6216; Santa Cruz Biotechnology) and secondary antibody rabbit anti-goat Ig/HRP (1:5,000, no. P0160; Dako).

### Intracellular staining.

293T cells were transduced by the CRNKL1 targeting or nontargeting CRISPR lentivectors at an MOI of 4. Two days later, the transduced cells were superinfected with VSV-G-pseudotyped HIV.r^−^e^−^ at an MOI of 1. For intracellular staining, 1.0 × 10^6^ cells were fixed at 2 days postsuperinfection using 2% paraformaldehyde (PFA) (room temperature, 30 min) and then permeabilized with 0.5% saponin in FACS buffer (room temperature, 10 min). Cells were subsequently stained with 100 µl anti-p24 primary antibody (7 ng/µl in permeabilization buffer; 4°C, 1 h), followed by washing with 1 ml permeabilization buffer two times. Staining with secondary antibody was done with 100 µl rat anti-mouse IgG1/allophycocyanin (APC) conjugated (no. 17-4015-82; eBioscience) (1:500 dilution in permeabilization buffer; 4°C, 1 h), followed by washing two times.

### Lentiviral vector preparation, infection, and titration.

Lentiviral vector particles were produced by transfection of 293T cells with the plasmids mentioned above. HIV-1 proviruses were packaged with a third-generation packaging system (Hgp^syn^, pcRev, pcTat, and pHiT-G) (27, 84, 85). pWPXL, pWPXL-Rev, CRISPR-KO constructs, and a pool of the two GeCKO v2 plasmid A and B sublibraries were packaged with a second-generation packaging system (pPAX2 and pMD2.G) (48, 86) for higher infectious titers. At 48 h after transfection, the supernatants from transfected 293T cells were cleared and then filtered through 0.45-μm filters. Viral stocks were aliquoted and stored under −80°C. Two batches of the GeCKO v2 lentiviral vector library were prepared. HiSeq NGS analyses of sgRNA representation in cells transduced with both batches showed an almost 100% identification of the sgRNAs encoded by the two GeCKO v2 plasmid sublibraries.

Jurkat, J-dual#3, and J-dual#6 cells were infected with the lentiviral vectors or the lentiviral vector library by spinoculation. A total of 1.5 million cells were infected with 1 ml vector preparation, in the presence of 8 μg/ml Polybrene (Sigma-Aldrich), at 2,000 rpm, for 3 h, at 33°C. After spinoculation, the supernatants were removed and the cells were continued to be cultured.

VSV-G-pseudotyped HIV.R^+^e^−^, HIV.r^−^e^−^, HIV.r^−^e^−^DsRed, and HIV-dual-GT vector particles were titrated on TZM-bl cells as previously described (63). The titers were 5.8 × 10^6^ and 5.1 × 10^6^ TU/ml for two independent titrations of HIV.R^+^e^−^, 4.3 × 10^6^ and 5.7 × 10^6^ TU/ml for two independent titrations of HIV.r^−^e^−^, 6.1 × 10^6^ and 5.4 × 10^6^ TU/ml for two independent titrations of HIV.r^−^e^−^DsRed, and 6.4 × 10^5^ and 5.0 × 10^5^ TU/ml for two independent titrations of HIV-dual-GT. VSV-G-pseudotyped HIV-dual-GT vector particles were also titrated on Jurkat cells. The lentiviral reporter vector was serially diluted to 1:1, 1:2, 1:4, 1:8, and 1:16. The infection was done by the spinoculation method. At 48 h postinfection, cells were analyzed by flow cytometry to determine the percentage of DsRed-positive cells in each dilution. Vector titers on Jurkat cells were calculated as follows for all dilutions: (initial cell number) × (percentage of DsRed-positive cells) × (dilution factor). The highest value resulting from these dilutions was taken as the vector titer on Jurkat cells. The determined titers for two independent titrations of HIV-dual-GT were 4.9 × 10^5^ and 6.9 × 10^5^ TU/ml. The individual CRISPR-KO vectors and pWPLX-Rev, as well as the GeCKO v2 lentiviral vector library, were titrated on J-dual reporter cells and parental Jurkat cells. The cells infected by the serially diluted virus were grown for 24 h and then divided into two equal aliquots. One aliquot was then cultured in 2 µg/ml puromycin, the other without puromycin. After 30 h of growth, viable cells were counted by trypan blue staining. The transduction percentage was calculated in the following way: [(number of living cells in treated population) × 100]/(number of living cells in untreated population). For the GeCKO v2 lentiviral vector library, the transduction percentage was multiplied by the number of cells exposed to calculate the titer for the different dilutions of the library. The highest value obtained from the different dilutions of the same batch of the lentiviral vector library was taken as its titer. The titers were 1.3 × 10^7^ TU/ml for the first batch and 2.2 × 10^6^ TU/ml for the second batch.

### Flow cytometry analysis and cell sorting.

Flow cytometry analyses were done using the BDLSRII flow cytometer (BD Biosciences). FACS data were analyzed by the FlowJo software. Cell sorting was done on a MoFlo XDP (Beckman Coulter) at the FACS core facility of the medical faculty.

### Next-generation sequencing of sgRNA libraries.

Genomic DNA was extracted from the sorted or control cells using either the Quick-DNA minikit or the Quick-DNA midi plus kit (Zymo Research). The integrated lentiCRISPRv2 vector sgRNA sequences were amplified through three rounds of PCR using the *Taq* DNA polymerase S (high specificity) (Genaxxon Bioscience). All obtained genomic DNA was used as a template in parallel reactions in PCR1, with the following cycling conditions: 94°C for 2 min, then 18 cycles of 95, 55, and 70°C for 15, 20, and 45 s, respectively, and a final extension at 70°C for 3 min. PCR1 reaction mixtures of respective cell samples were then combined, and 10 μl of the pooled PCR1 product was used as a template in PCR2, consisting of 95°C for 2 min, then 15 cycles of 95, 60, and 72°C for 15, 20, and 30 s, respectively, and a final extension at 72°C for 3 min. In the indexing PCR3, 10 ng of purified PCR2 product was subjected to 95°C for 2 min, then 8 cycles of 95, 59, and 72°C for 15, 20, and 30 s, respectively, and a final extension at 72°C for 3 min. The primer sequences and primer combinations used for each sample at each step are presented in Tables S1B and C. After PCR2 and PCR3, PCR products were purified by the AMPure XP PCR-cleanup kit (Beckman Coulter); DNA concentrations were measured by the Qubit Quant-iT dsDNA HS assay kit (Thermo Fischer Scientific). Manipulations followed the manufacturers’ instructions. DNA libraries were sequenced on Illumina MiSeq (for enriched samples) or Ilumina HiSeq 2500 (for unselected control) instruments. NGS readouts were subjected to MAGeCK analysis (49).

### Cell fractionation, RNA isolation, and RNA quantification.

The cell fractionation protocol has been described previously (87). The plasma membrane was lysed with chilled NP-40 buffer consisting of 10 mM HEPES (pH 7.8 adjusted by KOH), 10 mM KCl, 20% glycerol, and 0.25% NP-40. Dithiothreitol (DTT) was added to a final concentration of 1 mM just before use. After incubation on ice for a maximum of 2 min, the supernatant consisting of the cytoplasmic fractions was collected after centrifugation at 400 × *g*, for 5 min, at 4°C. Cytoplasmic RNA was extracted by the TRIzol (Thermo Fisher Scientific)-chloroform (Sigma-Aldrich) method (88) or by the Direct-zol RNA miniprep plus kit (Zymo Research). RNA from the whole-cell extracts was purified using the RNeasy kit (Qiagen). RNA extracted by TRIzol-chloroform or the RNeasy kit was treated with DNase I (NEB) to remove DNA contamination. Under the protection of 5 mM EDTA, DNase I was inactivated at 75°C for 10 min. For the Direct-zol RNA miniprep plus kit-purified RNA, DNA contamination was removed by the in-column DNA digestion reagents supplied. The total RNA concentration was measured by the Qubit Quant-iT RNA HS assay kit (Thermo Fisher Scientific) according to the manufacturer’s instructions.

Reverse transcription-PCRs (RT-qPCRs) for quantification of HIV-1 US and FS RNAs and cellular GAPDH pre-mRNAs were done with the QuantiTect SYBR green RT-PCR kit (Qiagen) and performed essentially as described previously (62, 89). Using the Applied Biosystems 7500 real-time PCR machine, cycling conditions were as follows: for HIV-1 US RNA, 95°C for 10 s for denaturation, 65°C for 60 s for annealing, and 72°C for 30 s for elongation and fluorescence detection; for HIV-1 FS RNA, 95°C for 10 s for denaturation, 64°C for 15 s for annealing, 72°C for 15 s for elongation, and 81°C for 30 s for fluorescence detection.

RT-qPCRs for relative quantification of intron 6-retaining RPL10 mRNAs and intron 1-retaining C19orf53 mRNAs were done with primers given in Table S1D. Cycling conditions for both were as follows: 95°C for 10 s for denaturation, 50°C for 60 s for annealing, and 72°C for 60 s for elongation and fluorescence detection.

### Calculation of the ratios of HIV FS to US RNA levels and fold changes.

To measure the effect of candidate genes and Rev on RNA splicing efficacy, we first determined the ratio of FS_whole_ to US_whole_ by dividing the FS copy numbers/ng extracted RNA by the US copy numbers/ng extracted RNA for each sample of each treatment group (knockdown or +Rev) and then calculated the mean of the ratios of replicates of each treatment group. The mean of the ratios of each treatment group was then divided by the mean of the ratios observed in matched control cells transduced with the NT1 or the GFP expression vector to calculate the fold changes given in Fig. 4C.

To measure the effect of candidate genes and Rev on the nuclear export efficacy (41) for HIV-1 US RNA for each treatment group, the mean US copy numbers/ng RNA extracted from the replicates of the cytoplasmic fractions were divided by the mean US copy numbers/ng RNA extracted from replicates of the whole-cell lysates. The ratio of each treatment group was then again divided by the ratio observed in controls cells transduced with the NT1 or the GFP expression vector to calculate the fold changes given in Fig. 4C.

Fold changes for cytoplasmic levels of US or FS RNA were calculated by dividing the mean US or FS copy numbers per ng of extracted RNA of each treatment group by the mean derived from the control groups.

### RNA immunoprecipitation.

Nuclear fractions from 2 × 10^7^ transfected and infected 293T cells were lysed by 600 µl of prechilled NP-40 lysis buffer (50 mM HEPES-KOH at pH 7.4, 150 mM KCl, 2 mM EDTA, 0.5% [vol/vol] NP-40, 0.5 mM DTT, Roche complete EDTA-free protease inhibitor cocktail, 40 U/ml Promega RNasin RNase inhibitor) while incubated on ice for 5 min. Lysates were cleared by centrifugation (16,000 relative centrifugal force [RCF], 4°C, 15 min). A 200-µl aliquot of the supernatant was used to quantify the amount of input RNA. Another 200-µl aliquot of the supernatant was incubated with 20 µl protein G Dynabeads (Invitrogen) precoated with 1 µg of anti-myc tag antibody or the same amount of an IgG2a isotype control antibody (Southern Biotech) respectively. RNP binding was performed overnight at 4°C. Washing was done with prechilled immunoprecipitation (IP) wash buffer (50 mM HEPES-KOH at pH 7.4, 150 mM KCl, 2 mM EDTA, 0.05% [vol/vol] NP-40, 0.5 mM DTT, complete EDTA-free protease inhibitor cocktail, 40 U/ml Promega RNasin RNase inhibitor) for six times with the aid of a magnetic rack (Invitrogen). Proteins bound to the beads were recovered from 10% of the beads by heat denaturation and characterized by Western blot analyses. RNA associated with the remaining 90% of the beads was purified by the TRIzol method. Input RNA and bead-associated RNA were analyzed by RT-qPCR.

### RNA-seq.

Cytoplasmic RNAs were extracted from the sorted BFP-positive or unsorted control J-dual#3 cells with the Direct-zol RNA miniprep plus kit. RNA qualities were controlled by the 2100 Bioanalyzer instrument (Agilent) (data not shown). Poly(A)-containing RNAs were enriched, fragmented, and reverse transcribed using the Illumina TruSeq stranded mRNA kit. Work flows for generating DNA libraries from each sample were performed essentially as described by the Illumina standard protocol (https://support.illumina.com/downloads/truseq-stranded-mrna-reference-guide-1000000040498.html). The libraries were sequenced on an Illumina HiSeq 2500 platform, and the reads were saved together with a quality score. Raw data were converted to reads including a quality score by Software bcl2fastq v2.17. Sequences matching Illumina TruSeq adapters were masked. Low-quality bases, poly(A) or poly(T) stretches, and masked bases were trimmed by Software fqtrim v0.9.5. Read quality was checked after sequencing and after base trimming by Software fastqc v0.11.8. For the viral genome, reads were aligned to HIV-dual-GT using hisat2 (90). Read counting for the different features was performed using samtools (91) and regtools (92).

For splicing and differential expression analysis, reads were aligned to the human transcriptome using RSEM v1.2.20 (93) and Bowtie v1.1.2 (93) as the read alignment program using default parameters. Differential expression analysis was performed by DESeq2 v1.18.1 (93) using the RSEM output for read counts per gene. We considered genes that showed an absolute log_2_-transformed fold change greater than 1 and an adjusted *P* value (*P*_adj_) of less than 0.1 as differentially expressed. To exclude low-expression genes, we considered only those with a mean number of transcripts per kilobase million (TPM) greater than 1 in either untreated or knockdown samples. Gencode v19 annotation was used in all steps of the analysis, including in the definition of protein coding genes. The read mapping profiles were exhibited via the Integrative Genomics Viewer (IGV) v2.3 (94). Differential splicing analysis was performed with SUPPA (71) using TPM values from the RSEM transcript isoform output. For PSI calculation, events were required to pass the expression filter of TPM greater than 1 (-f 1 parameter). We considered splicing events with *P*_adj_ values lower than 0.01 and absolute ΔPSI values greater than 0.25 as differentially included or excluded. Polypyrimidine track length and branch point scores were calculated using BPfinder (95). Splice site strength was calculated using MaxENT (96) (https://hollywood.mit.edu/burgelab/software.html).

### Data availability.

The raw and processed data of RNA-seq are accessible via GEO (Gene Expression Omnibus) accession number GSE144347.
