# Supplementary figures and images for: CRNKL1 Is a Highly Selective Regulator of Intron-Retaining HIV-1 and Cellular mRNAs
Source: mBio. 2021 Jan 19;12(1):e02525-20. doi: 10.1128/mBio.02525-20 (PMC7845644; doi:10.1128/mBio.02525-20)

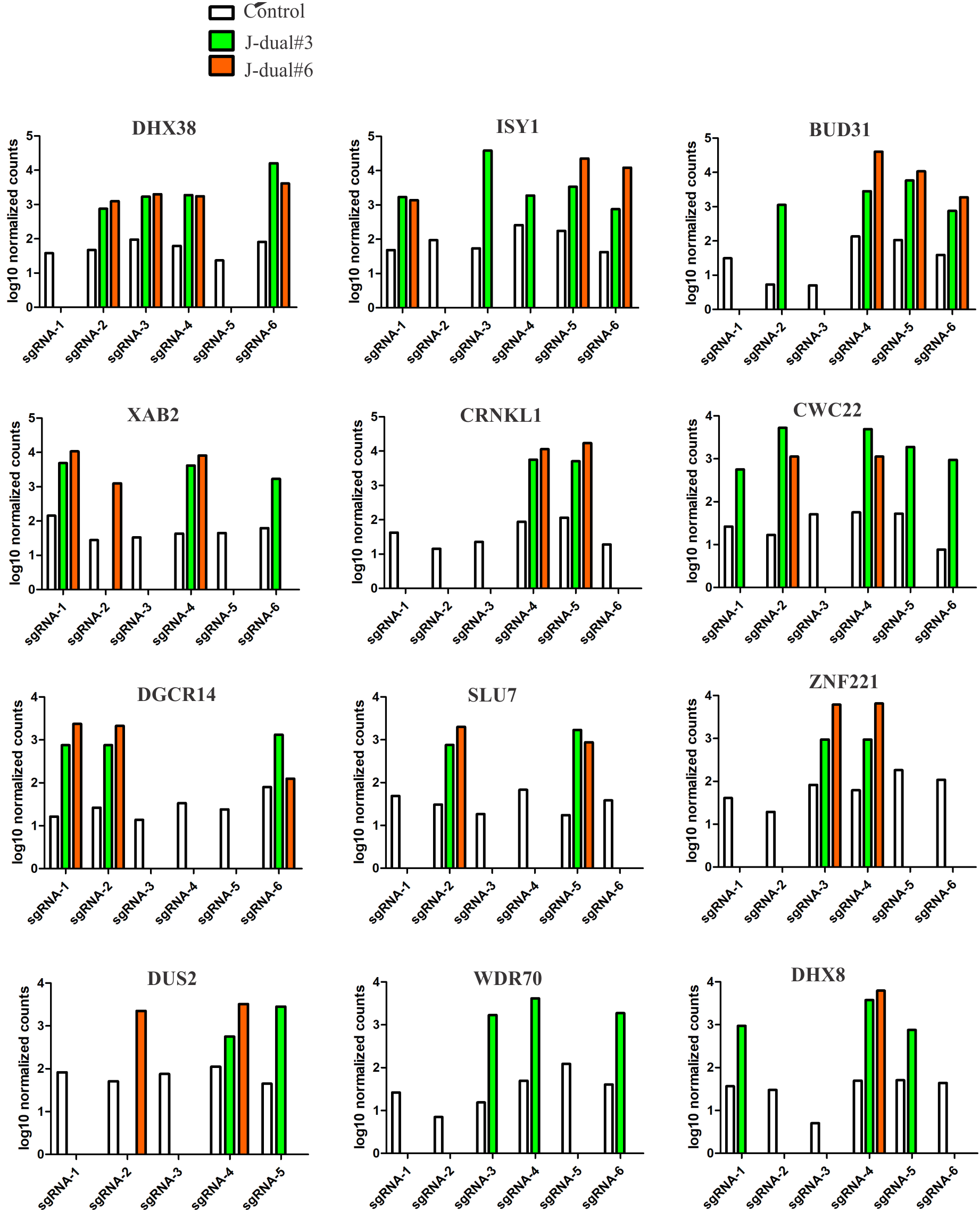

Supplement: FIG S1 [file mBio.02525-20-sf001.tif]

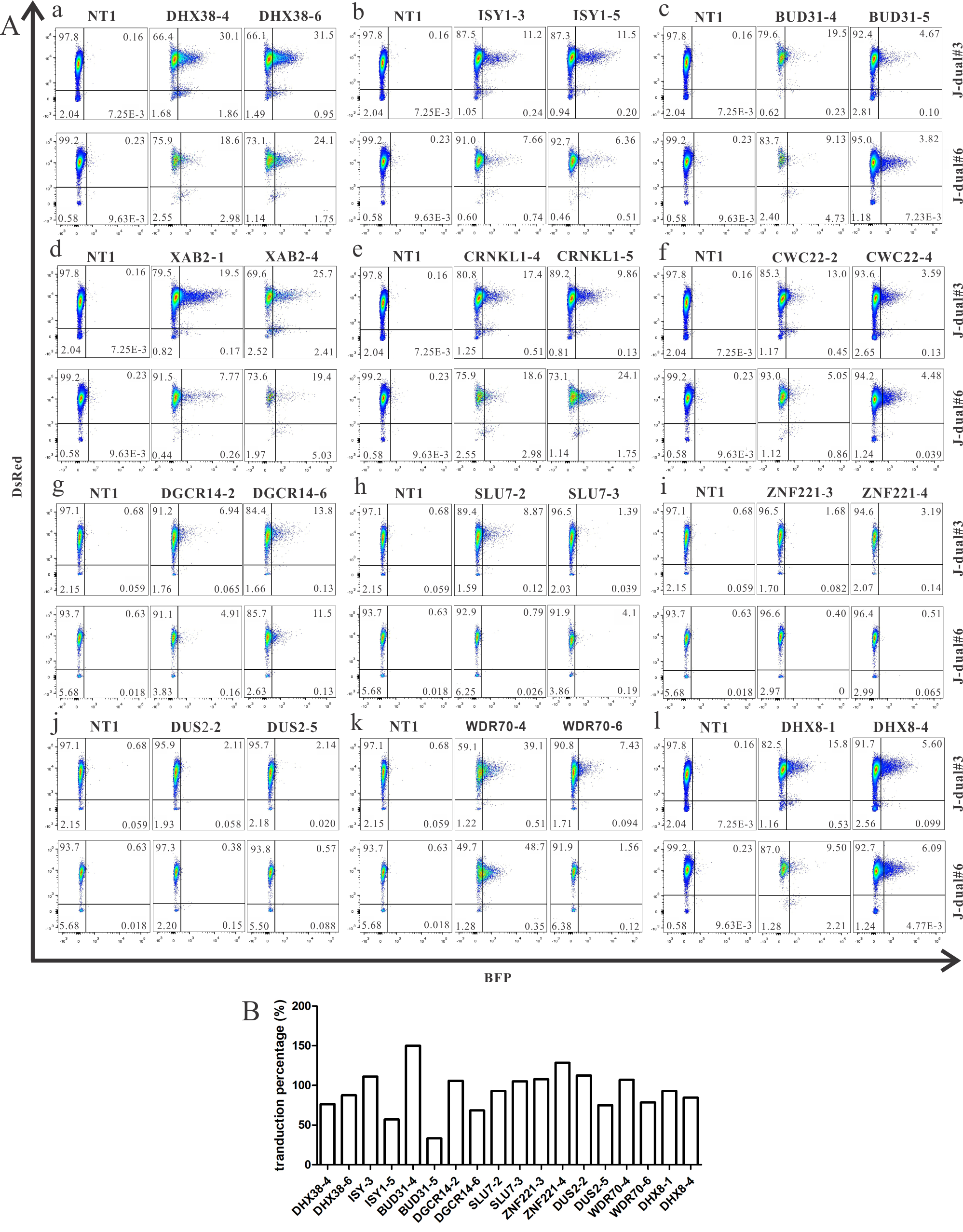

Supplement: FIG S2 [file mBio.02525-20-sf002.tif]

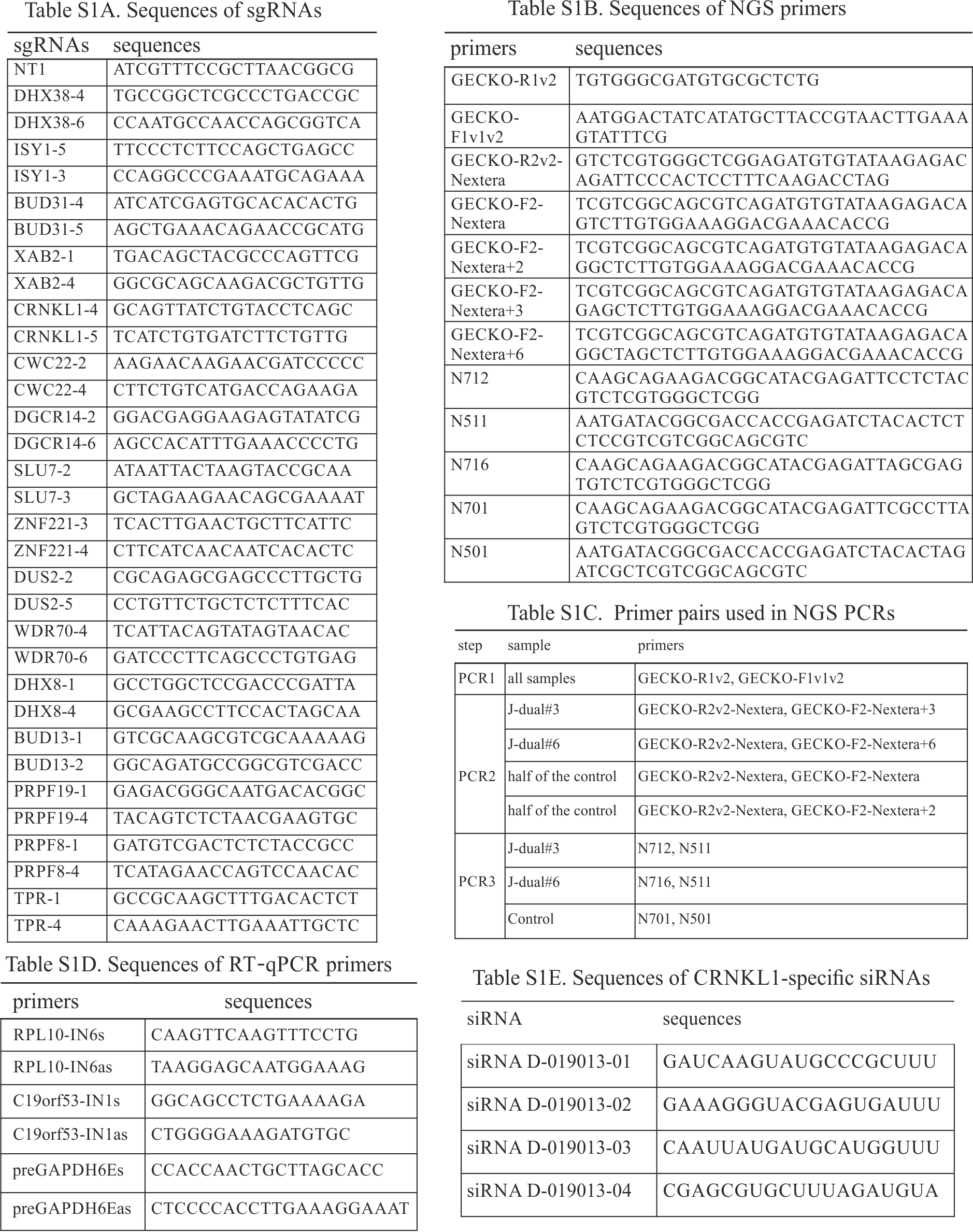

Supplement: TABLE S1 [file mBio.02525-20-st001.tif]

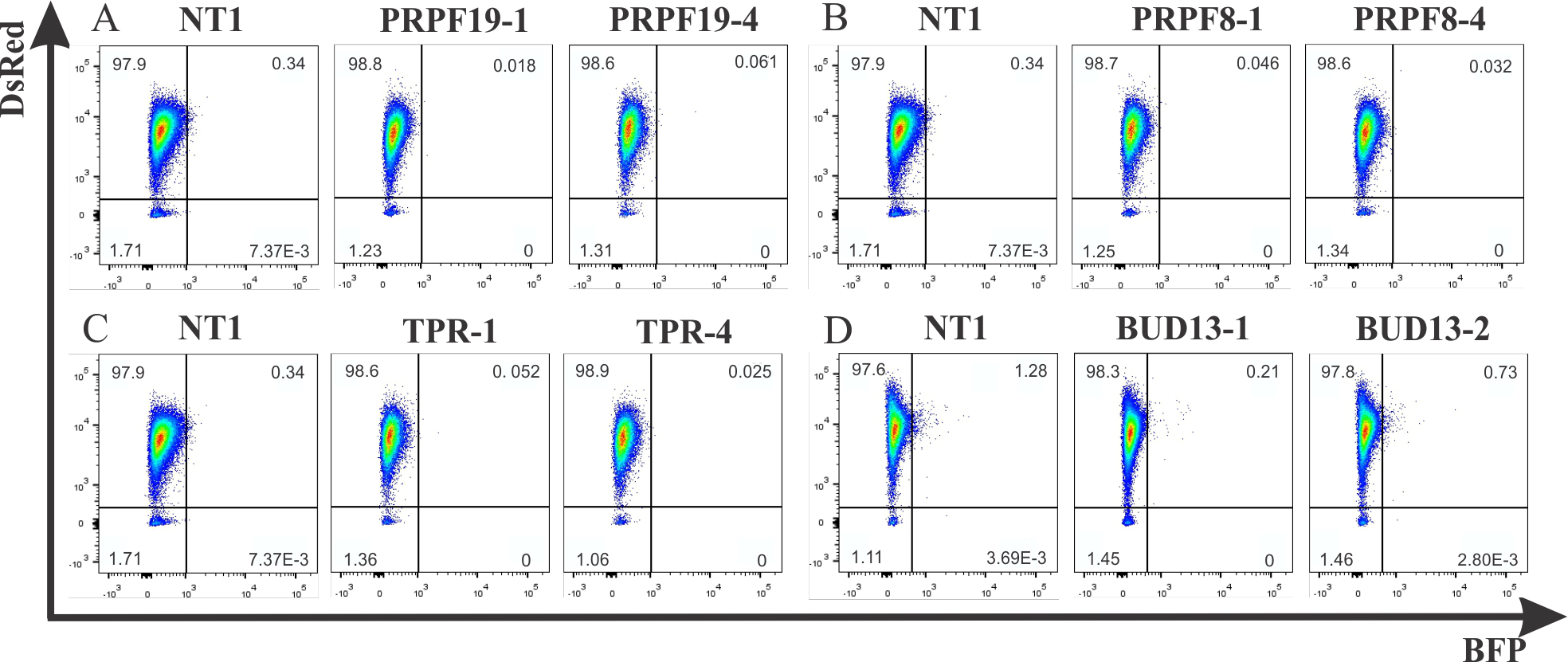

Supplement: FIG S3 [file mBio.02525-20-sf003.tif]

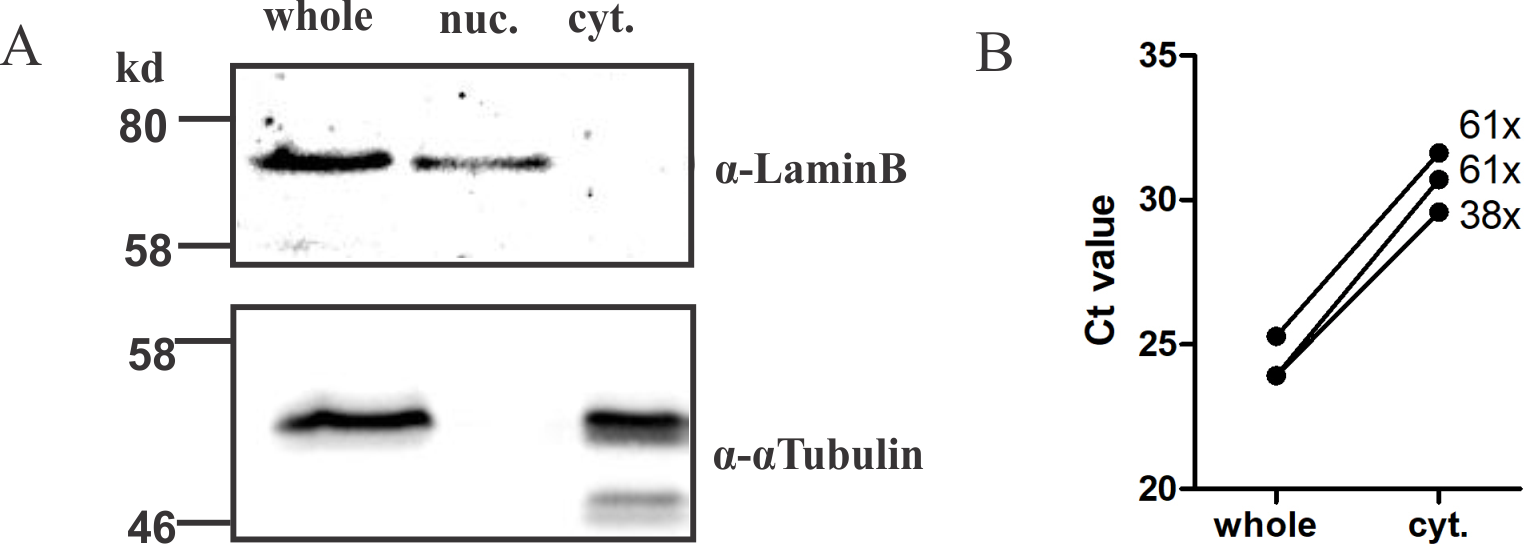

Supplement: FIG S4 [file mBio.02525-20-sf004.tif]

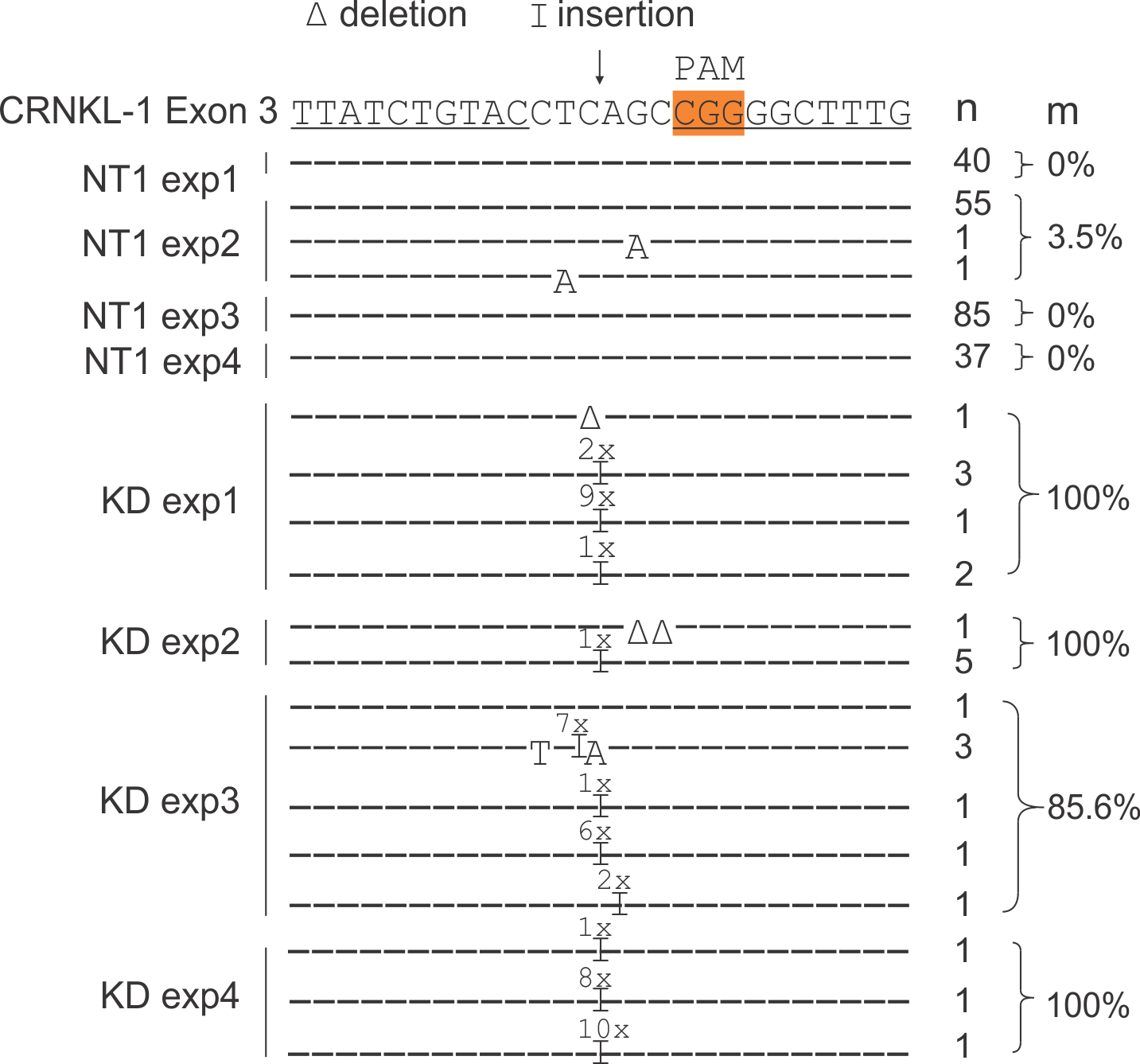

Supplement: FIG S5 [file mBio.02525-20-sf005.tif]

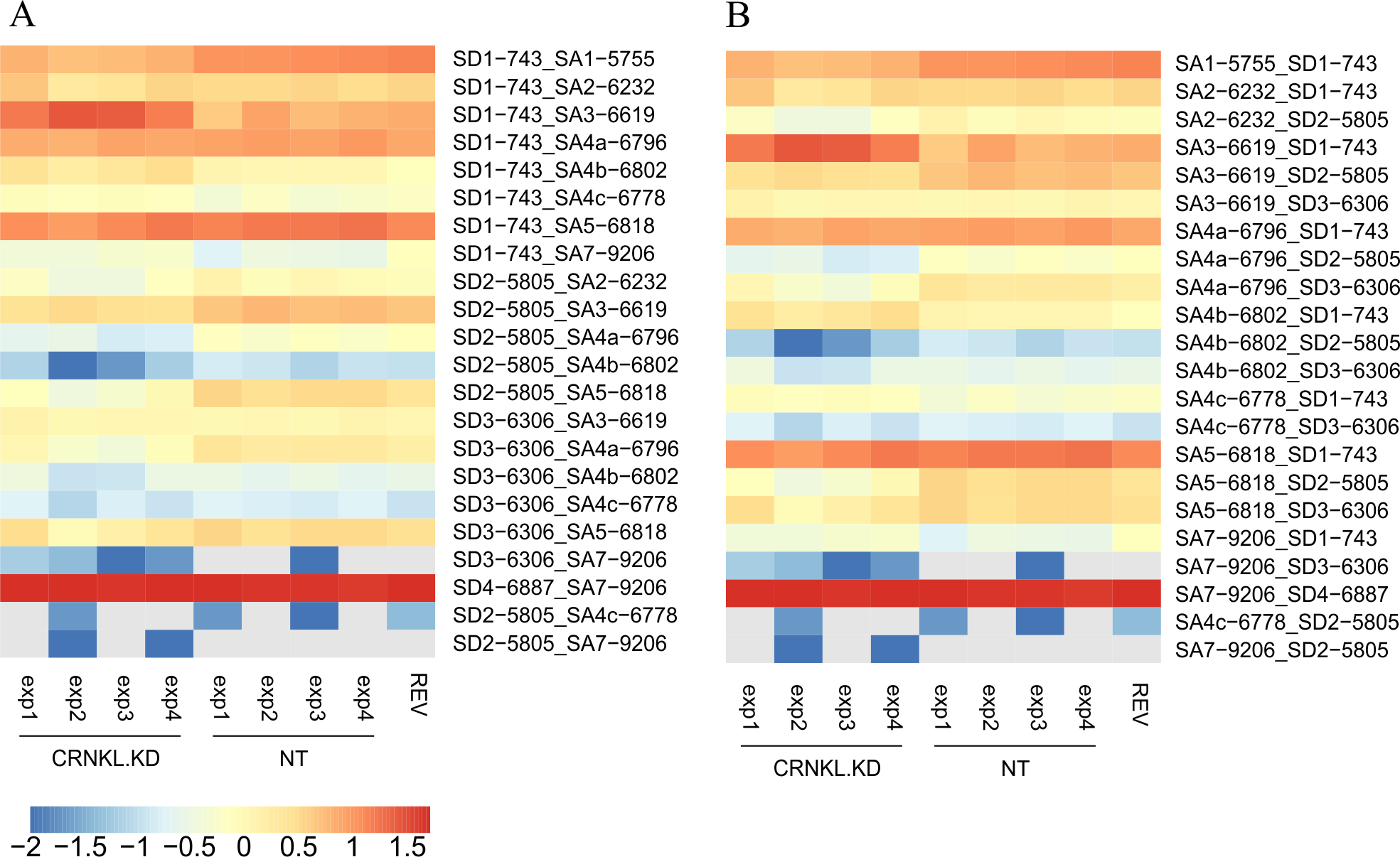

Supplement: FIG S6 [file mBio.02525-20-sf006.tif]

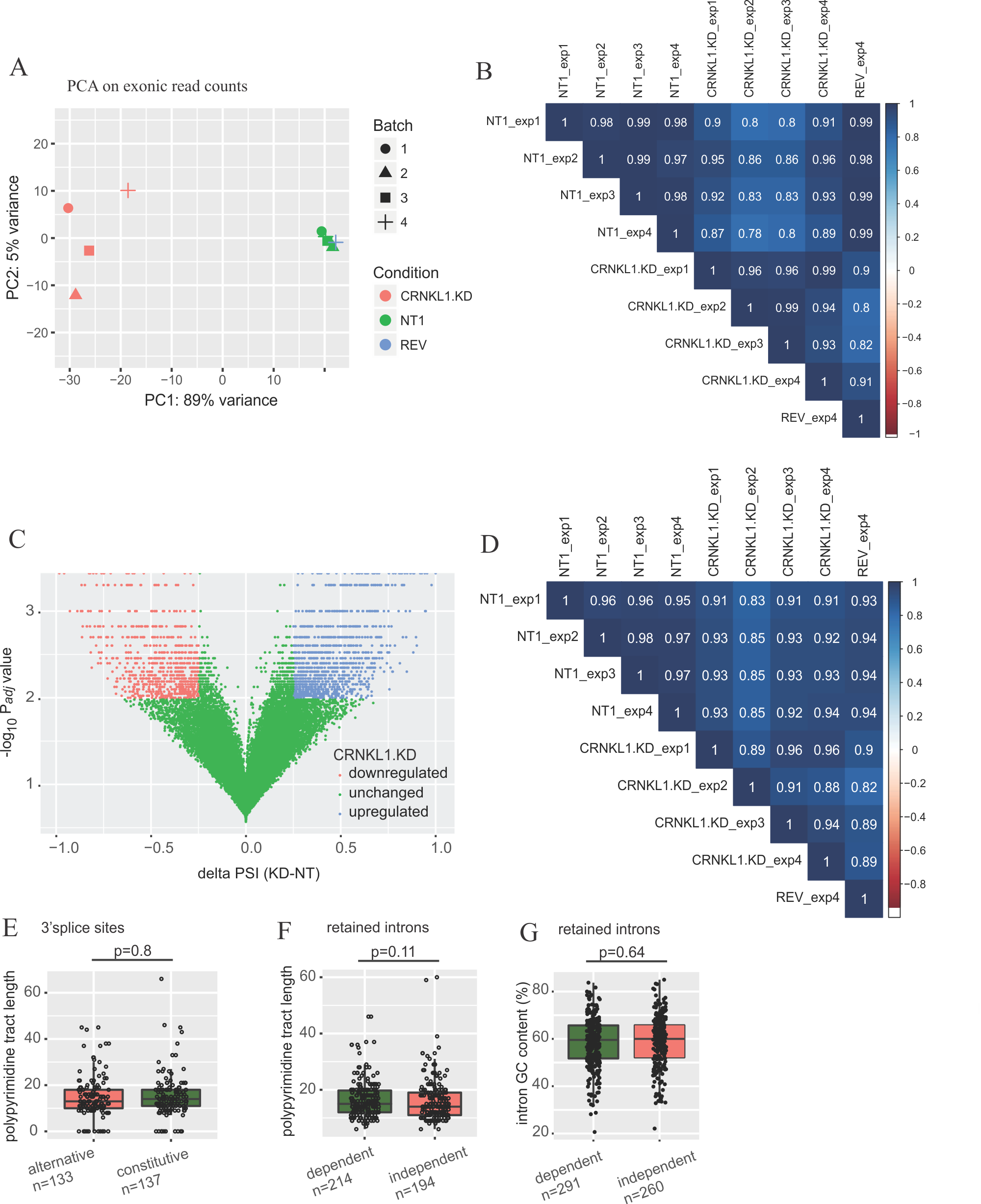

Supplement: FIG S7 [file mBio.02525-20-sf007.tif]

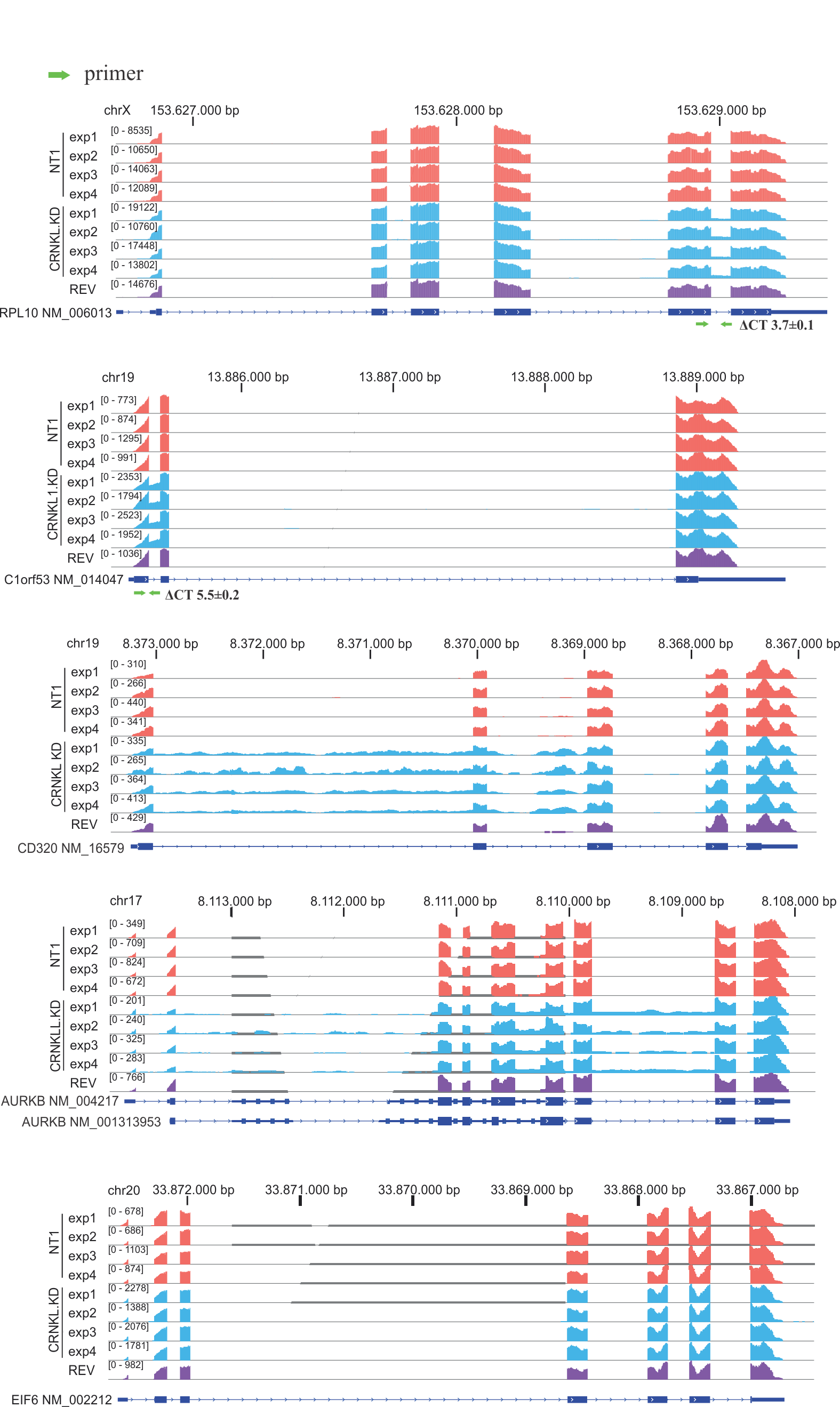

Supplement: FIG S8 [file mBio.02525-20-sf008.tif]
